# Linking mPFC circuit maturation to the developmental regulation of emotional memory and cognitive flexibility

**DOI:** 10.7554/eLife.64567

**Published:** 2021-05-05

**Authors:** Cassandra B Klune, Benita Jin, Laura A DeNardo

**Affiliations:** 1 Physiology Department, David Geffen School of Medicine, UCLALos AngelesUnited States; 2 Neuroscience Interdepartmental Graduate Program, UCLALos AngelesUnited States; 3 Molecular, Cellular and Integrative Physiology Graduate Program, UCLALos AngelesUnited States; New York UniversityUnited States; University of Texas at AustinUnited States

**Keywords:** mPFC, development, circuit, synapse, cognitive flexibility, memory

## Abstract

The medial prefrontal cortex (mPFC) and its abundant connections with other brain regions play key roles in memory, cognition, decision making, social behaviors, and mood. Dysfunction in mPFC is implicated in psychiatric disorders in which these behaviors go awry. The prolonged maturation of mPFC likely enables complex behaviors to emerge, but also increases their vulnerability to disruption. Many foundational studies have characterized either mPFC synaptic or behavioral development without establishing connections between them. Here, we review this rich body of literature, aligning major events in mPFC development with the maturation of complex behaviors. We focus on emotional memory and cognitive flexibility, and highlight new work linking mPFC circuit disruption to alterations of these behaviors in disease models. We advance new hypotheses about the causal connections between mPFC synaptic development and behavioral maturation and propose research strategies to establish an integrated understanding of neural architecture and behavioral repertoires.

## mPFC circuits: prolonged maturation and targets for early intervention

In the rodent, the medial prefrontal cortex (mPFC) comprises the anterior cingulate cortex (ACC), the prelimbic cortex (PL), and the infralimbic cortex (IL), which each have distinct connectivity and functional properties. As a whole, they are densely interconnected with other cortical association areas, the limbic system, midline thalamic nuclei, and an array of midbrain and brainstem nuclei with unique behavioral functions. Through these diverse inputs and outputs, mPFC plays a key role in decision making, memory, social interactions, mood, and cognition (Kolb and Nonneman, 1978; Kolb et al., 1974; Nonneman et al., 1974). In this review, we focus on cognitive flexibility and emotional learning and memory in both the aversive and appetitive domains. Like many mPFC-dependent functions, the encoding and expression of emotional memories, as well as cognitive flexibility, are developmentally regulated, emerging, and maturing during early life and adolescence when the prefrontal cortex is still developing (Kalsbeek et al., 1988; Van Eden and Uylings, 1985; Lewis, 1997; Kolb et al., 2012).

**Figure 1.**
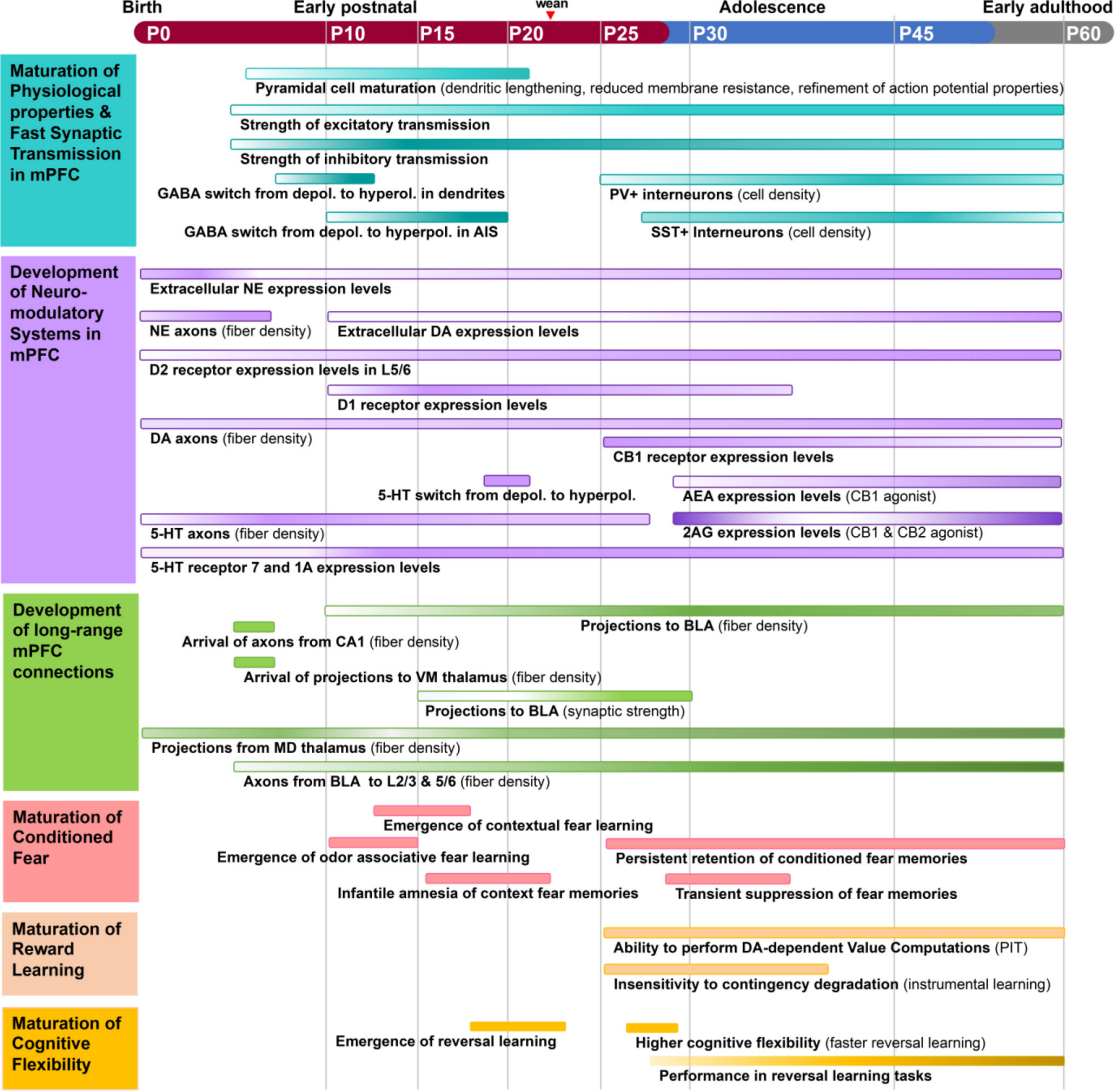
Timeline of major events during rodent medial prefrontal cortex (mPFC) development. The structural and functional organization of mPFC circuitry is largely established in early postnatal development and then refines into early adulthood. Adolescence is marked by enhanced bidirectional innervation of mPFC, amygdala, and neuromodulatory centers. Inhibitory neurotransmission increases from P15–P60, with major changes in the excitatory/inhibitory ratio of synaptic transmission in adolescence. Aspects of mPFC circuit development align with the maturation of cognitive behaviors. The ability to perform reversal learning and contextual fear learning emerges just prior to adolescence and remains highly malleable until at least mid-adolescence. Each process is represented as a colored bar, with the gradient of color intensity (low to high) marking initiation, peak, and decline of the process where applicable. Of note, bars representing the magnitude of axonal innervation usually begin at the earliest point reported in the literature, but do not remove the possibility of earlier innervation.

From birth until early adulthood, mPFC cells and circuits undergo changes in their physiological properties and the strength of connectivity with distant brain regions including limbic and neuromodulatory centers (Kolb et al., 2012; Figure 1). This prolonged period of development may be necessary because establishing complex behaviors requires an extended interaction with the environment. However, it opens a long window during which mPFC is vulnerable to disruption. Indeed, numerous mPFC-dependent behaviors are altered in neuropsychiatric disorders that emerge during childhood and adolescence including anxiety disorders, impulse control disorders, depression, schizophrenia, and autism spectrum disorder (ASD) (Schubert et al., 2015). Although mPFC dysfunction is strongly implicated in these diseases, we are only beginning to understand the mechanisms that drive the milestones in mPFC neurodevelopment and support the maturation of adaptive behaviors.

Below we discuss converging cross-species evidence from genetic, neuroanatomical, and neurophysiological studies that reveal continuous remodeling of mPFC cells, synapses, and circuits throughout healthy development, with dramatic changes occurring from the end of the juvenile period through adolescence. We focus on the rodent brain because it is a genetically tractable system that can establish causal links between circuits and behavior. We highlight new research, presenting it chronologically according to rodent development. In the juvenile period, mPFC neurons undergo increases in excitability and synaptic strengthening while long-range connections form and oscillatory rhythms emerge. We postulate that these changes may underlie developmental changes in the ability to form long-term fear memories. In adolescence, we focus on the maturation of inhibitory and neuromodulatory circuits. During adolescence, dramatic increases in mPFC synaptic inhibition align with behavioral changes in conditioned fear, reward learning, and reversal learning. At the same time, the staggered maturation of the monoamine and endocannabinoid (EC) systems shapes mPFC development in ways we are just beginning to understand. We consider how these processes may be causally related. New research on key disease-relevant genes underscores mPFC inhibitory microcircuits, neuromodulatory systems, and long-range connectivity as some of the most vulnerable elements of developing mPFC circuits. This new work highlights an urgent need to refine our understanding of the mechanisms governing mPFC circuit assembly and to establish causal links between the maturation of mPFC circuits and behavior.

We define postnatal day (P)0–P27 as the juvenile period, P28–P48 as adolescence, and P49–P60 as young adulthood. Each period is marked by milestones in behaviors, sexual maturity, and the structure and function of mPFC cells and circuits ([Bibr bib29][Bibr bib156]; Agoglia et al., 2017; Brust et al., 2015). Others have defined the adolescent period in rat as P28–P42 based on behaviors such as increased risk-taking and social play, inclusion of peak growth spurts, and the emergence from the parental nest in the wild (Spear, 2000). Still, this age range is noted as conservative and its margins can significantly vary per individual and sex. For example, female rats become sexually mature between P32– and P34, but male rats typically mature much later at P45–P48 (Lewis et al., 2002). Studies on fear learning, addiction, reward, and social behaviors define adolescence from as young as P28 to as old as P60, though they center on the range of P28–P48 (Hefner and Holmes, 2007; Adriani et al., 2004; Simon and Moghaddam, 2015; Bell, 2018). Adulthood is generally considered to begin between P60 and P70 in rats (McCutcheon et al., 2009). We integrated this literature to establish our definitions for rats and mice.

By aligning key events during mPFC development with transition points during the maturation of complex behaviors, this review highlights missing links between genes, circuits, and behaviors. We propose that the maturation of the component parts of mPFC circuitry subserves the ontogeny of complex behaviors, with the behavioral functions of mPFC updating during transitions between critical windows of development. We put forth novel hypotheses that can be tested by using cutting-edge viral-genetic tools to link circuit-level and behavioral changes in the developing brain. New research that addresses these questions could ultimately pinpoint developmental vulnerabilities in mPFC circuits that give rise to pathological states.

## The juvenile period: P0–P27

From birth until the week after weaning (P27), neurons in the rodent mPFC undergo a series of remarkable anatomical and physiological transformations while long-range projections establish their connectivity with distant targets. Patterns of oscillatory activity, which can indicate the coordinated activity of mPFC with distal brain regions and can facilitate long-range information transfer, begin to appear. In this section, we align these milestones with evidence that mPFC becomes required for the expression of conditioned fear at the end of the juvenile period. We propose that the juvenile development of mPFC’s long-range connections, particularly those with the basolateral amygdala (BLA), is necessary for mPFC’s ability to regulate conditioned fear. Furthermore, the immaturity of mPFC and its connections may contribute to the rapid forgetting of contextual fear memories that is observed prior to P24. Reward learning studies in juvenile rodents are scarce, likely because of challenges related to food restriction and operant training in young animals. As a result, this section will focus on how the development of mPFC in the juvenile period facilitates fear learning and memory.

During early postnatal development, pyramidal cells within the mPFC show morphological and functional changes characteristic of synapse development and maturation. During the first month of life, pyramidal cells in layer (L)3 and L5 in the mouse mPFC undergo dendritic lengthening and increases in spine density (Kroon et al., 2019). The pyramidal cell growth in mPFC is accompanied by increases in the speed and amplitude of action potentials and decreased input resistance consistent with increases in ion channel density (Kroon et al., 2019). Between P17 and P24, markers of excitatory and inhibitory synapse maturation significantly increase in rat mPFC (Jia et al., 2018). In general, juvenile (P24–P28) pyramidal neurons in mPFC of mice have more dendritic spines and show more spine turnover than in adults (P64–P68) (Johnson et al., 2016). Thus, juvenile development is characterized by a robust period of synaptogenesis as well as synaptic pruning and refinement (Figure 1). For an in-depth review about changes in dendritic spines and synapse numbers during the juvenile period and adolescence, see Delevich et al., 2018.

During the first two postnatal weeks, mPFC neurons ramp up their spontaneous firing rates significantly (Brockmann et al., 2011). At P17, rats display significantly higher levels of the immediate early genes (IEG) *Arc*, *c-Fos,* and *Zif268* compared to P24 (Jia et al., 2018). IEGs are expressed rapidly in response to cellular events such as depolarization and are often used as markers of neuronal activity (Minatohara et al., 2015). In adults, basal levels of expression are low but IEGs are transiently induced in response to external stimuli (Minatohara et al., 2015). Thus, the increased IEG expression in early life may reflect immature regulatory mechanisms that eventually control neuronal activity in the adult brain including local inhibition and neuromodulation.

Throughout development, there is a gradual yet specific transition in the ratio of excitatory to inhibitory synaptic inputs per cortical layer. This layer specificity is already evident within the first postnatal month. By the second postnatal week, L3 pyramidal neurons exhibit more excitatory than inhibitory spontaneous synaptic events while L5 pyramidal neurons exhibit roughly equal amounts of each (Kroon et al., 2019). While these laminar differences persist until P30, it is not known to what extent they remain in adulthood. Given that mPFC layers have unique inputs and outputs (Gabbott et al., 2005; DeNardo et al., 2015) and display differential receptor expression (Radnikow and Feldmeyer, 2018), layer-specific differences may indeed persist into adulthood. Such spatiotemporal differences could ultimately establish discrete subcircuits that permit the mPFC to partake in several complex behaviors in a multifaceted manner. Further studies need to be done to elucidate the mechanisms of local mPFC circuit assembly in development.

In addition, oscillatory rhythms, which are critical for precise information flow between regions, begin to emerge in the mPFC as early as the first postnatal week. As early as P3, the rodent mPFC exhibits intermittent spindle-shaped field oscillations that are slower (mostly theta frequency), smaller, and less frequent than their counterparts in sensory cortices (Brockmann et al., 2011). By P5, short periods of low gamma frequency oscillation emerge superimposed on top of these spindles (Brockmann et al., 2011). Around P10, continuous oscillations emerge with theta as their dominant frequency. These changes occur along a slower developmental trajectory than in sensory cortices. Theta oscillations are dominant in mPFC-hippocampal communications in the adult brain, and mPFC oscillatory activities show strong coherence with hippocampal theta even in the first postnatal weeks. Interestingly, Granger causality analyses revealed that the hippocampus was a stronger driver of mPFC activity compared to vice versa, but only in the youngest animals studied (P6–P9) (Brockmann et al., 2011). This suggests that initially the hippocampus entrains network's activity in mPFC and that strengthening of descending influence from the mPFC to the hippocampus occurs later. In both humans and rodents, prefrontal theta is important in memory processes including working memory, fear memory, and reward memory (Backus et al., 2016; Lesting et al., 2013; Courtin et al., 2014; Hyman, 2010; Nishida et al., 2009).

**Figure 2.**
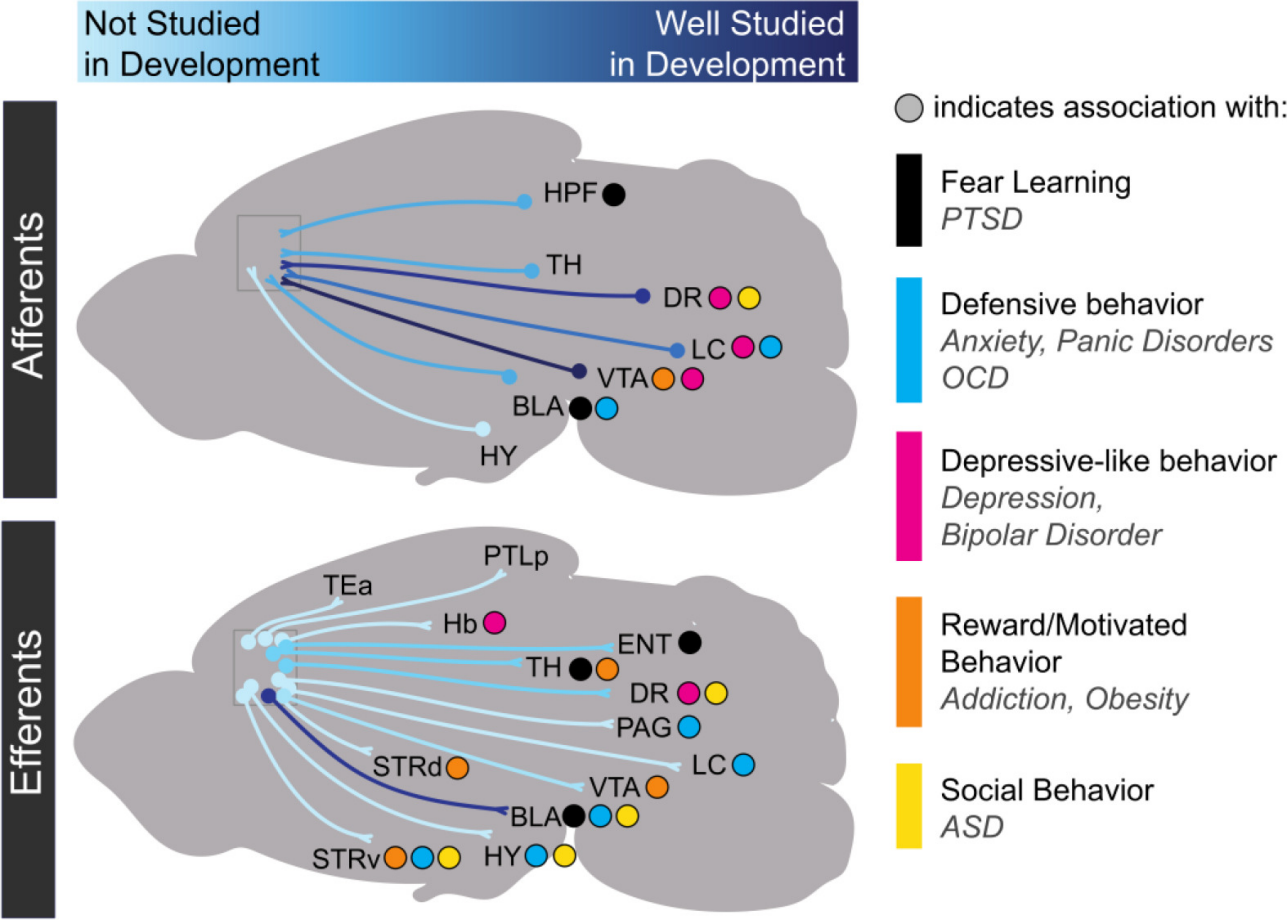
Progress in research on the development of medial prefrontal cortex long-range connectivity. Light blue indicates that a particular projection has not been studied in development while dark blue indicates that it has been relatively well-studied. Dots indicate behavioral repertoires and diseases associated with particular connections. HPF: hippocampal formation; TH: thalamus; DR: dorsal raphe nucleus; VTA: ventral tegmental area; LC: locus coeruleus; BLA: basolateral amygdala; HY: hypothalamus; TEa: temporal association area; PTLp: posterior parietal association area; Hb: habenula; ENT: entorhinal cortex; PAG: periaqueductal gray; STRd: dorsal striatum; STRv: ventral striatum.

Long-range synaptic connections, which support oscillatory rhythms in mPFC, form and strengthen throughout the early postnatal period. By P7, mPFC axons begin to innervate ventromedial (VM) thalamus (Hartung et al., 2016). mPFC axons begin to innervate the BLA around P10 and continue to increase in density until P30 (Arruda-Carvalho et al., 2017). mPFC sends projections to the lateral entorhinal cortex after P7 (Hartung et al., 2016). Afferent axons targeting mPFC arrive along a similar time course. By P16, amygdalar axons have begun to innervate L2 and L5 of mPFC in the PL and IL subregions (Cunningham et al., 2002). By P7, axons from the hippocampus, VM thalamus, and lateral entorhinal cortex have also reached PL where they play a critical role in the development of oscillatory activity (Hartung et al., 2016). Axons from mediodorsal (MD) thalamus are present by P1, decrease their density at P13, and then increase innervation into adulthood (Ferguson and Gao, 2014). Still, most studies of synaptic connectivity in developing mPFC long-range circuits have been purely anatomical or are lacking altogether. As such, there are a tremendous number of future directions in which researchers can examine the developmental time course and mechanisms of synapse formation between mPFC and long-range targets (Figure 2).

**Figure 3.**
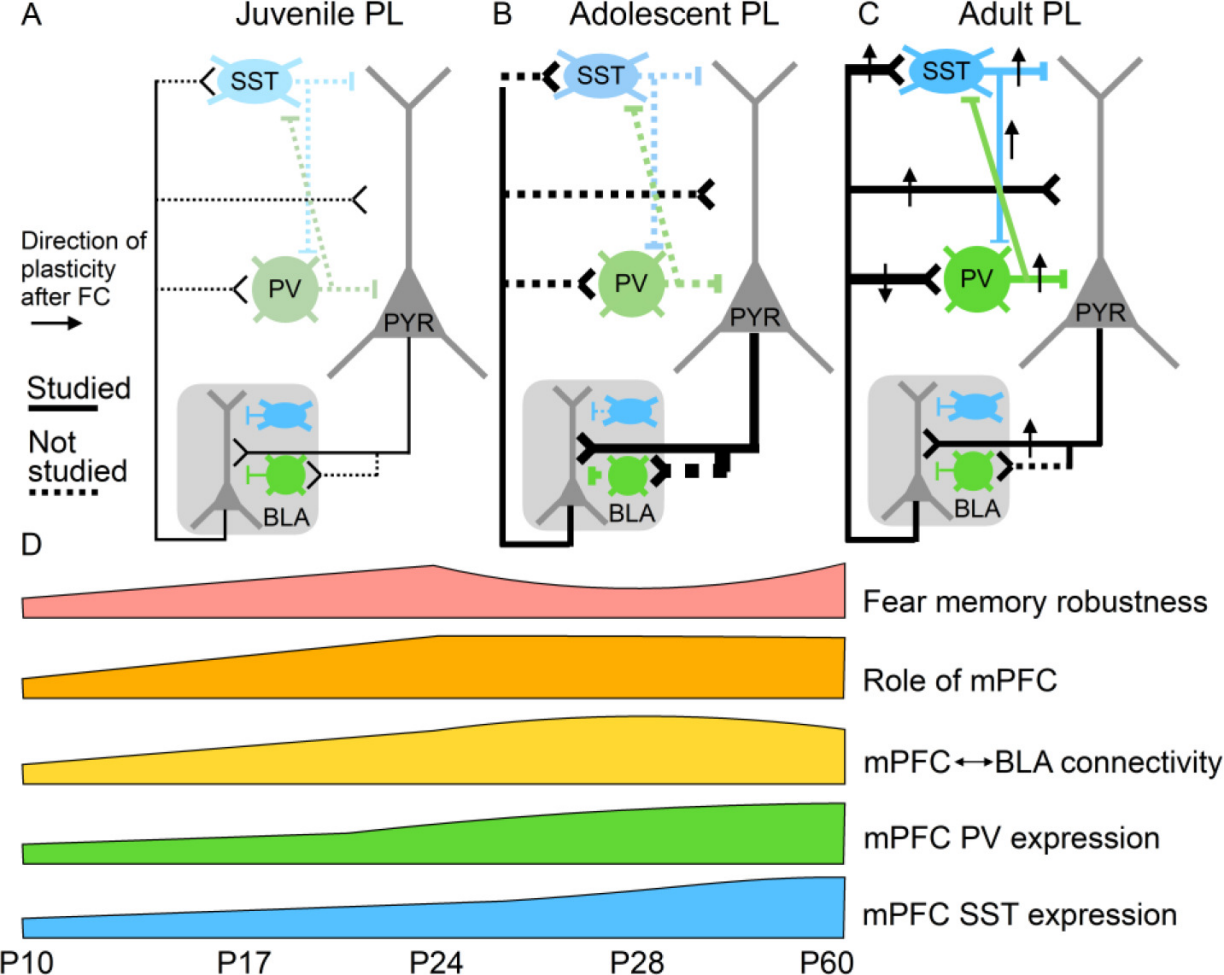
Potential relationships between prelimbic cortex-basolateral amygdala (PL–BLA) circuit assembly and the development of persistent fearful memories. (**A**) During juvenile period, weak connections between PL and BLA may contribute to infantile amnesia. (**B**) During adolescence, BLA axons continue to innervate PL, and there is a major increase in feed-forward inhibition in the PL projection to the BLA. In addition, parvalbumin-positive and somatostatin inhibitory interneurons, which are known to receive direct synaptic input from BLA, undergo physiological changes. Changes in inhibitory dynamics may contribute to the temporary suppression of fearful memories during adolescence. (**C**) In the adult, when fearful memories are robust and long-lasting, PL–BLA circuitry has stabilized in its mature form, with a slight refinement in the strength of the descending projection from PL to BLA, and the ascending projection from BLA to PL exhibiting stronger connections onto local interneurons than onto pyramidal cells.

mPFC connections with limbic centers and thalamic nuclei play key roles in a number of adaptive, disease-relevant behaviors including the expression of conditioned fear. It is thus likely that synaptic maturation in these pathways is necessary for the maturation of these behavioral functions. Changes in mPFC long-range connectivity with the BLA, which is essential for the learning and retrieval of emotional memories (LeDoux, 2009), may play a key role in the developmental regulation of long-lasting fear memories. Like humans, rodents exhibit infantile amnesia, meaning that memories formed early in life are short lasting, while those formed later in life persist (Ramsaran et al., 2019). This phenomenon has been modeled in rodents with contextual fear conditioning (CFC) (Akers et al., 2012; Akers et al., 2014) and inhibitory avoidance learning (IA) (Travaglia et al., 2016). In both tasks, rodents trained before the third postnatal week undergo rapid forgetting of learned fearful associations within a week. In contrast, rodents trained in the fourth postnatal week exhibit lasting memories of fearful associations (Akers et al., 2012; Akers et al., 2014; Travaglia et al., 2016). Although the causal role of the mPFC has not been explored in depth during CFC and IA in development, there is evidence that the expression of cued fear prior to P23 can occur independently of mPFC signaling (Li et al., 2012). P24 thus seems to be a turning point in the synaptic development of mPFC connections with the BLA, in the involvement of mPFC in associative fear memories, and in the development of persistent fearful memories, suggesting that these processes may be causally linked (Figure 3).

Importantly, in neuropsychiatric disorders, environmental disruptions and genetic mutations occurring in development may disrupt the early stages of wiring in mPFC inhibitory microcircuits and long-range connectivity. There is a rich body of literature demonstrating that early experience shapes mPFC cells and circuits and the behaviors that rely on them. These studies have been reviewed extensively elsewhere (Kolb et al., 2012; McEwen and Morrison, 2013; Nussenbaum and Hartley, 2019). There remain many open questions about the developmental time course and molecular mechanisms that control mPFC circuit assembly at juvenile stages, and the maturation of mPFC-dependent behaviors during the juvenile period is just beginning to be understood. Establishing links between neural and behavioral processes early in development will ultimately inform our understanding of early stages of disease progression, allowing for development of preventative interventions. Later, we discuss how mutations in disease risk genes cause convergent phenotypes in mPFC inhibitory circuits and long-range connectivity and regulate the organization of neuromodulatory systems, underscoring these circuit elements as some of the most vulnerable during disease progression. In the concluding paragraphs, we discuss how new studies can bridge the gaps between circuit maturation and juvenile behaviors using viral-genetic approaches.

## The adolescent period: P28–P48

Adolescence is a key period of maturation for fear and reward learning-related behaviors and cognitive flexibility in which animals employ unique behavioral strategies compared to their adult counterparts. Both human and rodent adolescents display heightened novelty-seeking and risk-taking that can broaden the repertoire of neural and behavioral states and, in doing so, assist individuals as they transition to independence from parental caretakers (Spear, 2000). Throughout the adolescent period, rodents gain the ability to form long-term fear memories, begin to display operant goal-directed behaviors, and improve their performance in tasks that require cognitive flexibility. In this section, we focus on three key aspects of development that likely underlie the maturation of these behaviors: (1) the development of inhibitory neurotransmission in mPFC, (2) the development of the monoamine neuromodulatory systems in mPFC, and (3) the development of the EC system in mPFC. Major developmental milestones in each of these areas are discussed and aligned with behavioral changes, while the lack of causal links and gaps in mechanistic understanding is highlighted.

## Development of inhibitory neurotransmission

**Figure 4.**
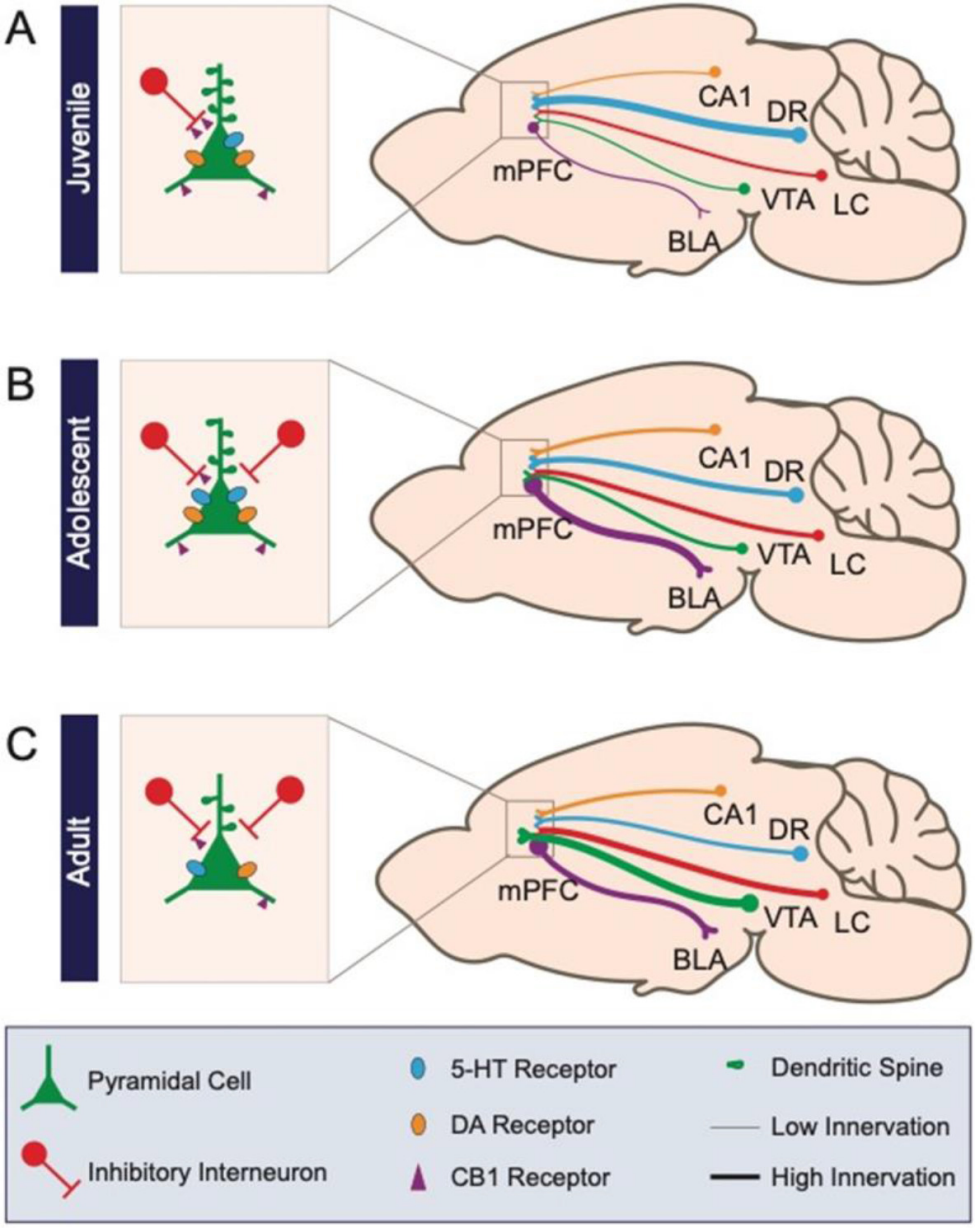
Schematic of prefrontal cellular and circuit changes throughout development. (**A**) The juvenile period is characterized by low-density anatomical connections and elevated spine density. (**B**) During adolescence, long-range connectivity strengthens along with local inhibitory circuits in medial prefrontal cortex. (**C**) In the adult, aspects of circuitry refine, including the density of dendritic spines and neuromodulatory receptors. Long-range axonal innervation density continues to increase between some regions. Numbers CA1: CA1 region of the hippocampus; DR: dorsal raphe nucleus; VTA: ventral tegmental area; LC: locus coeruleus; BLA: basolateral amygdala.

mPFC inhibitory interneurons undergo robust developmental changes in the adolescent period and are critical for the encoding and expression of fear memories in adulthood. During adolescence, the maturation of inhibitory circuits in mPFC may thus drive changes in fear memory expression. Here, we propose that changes in the number of parvalbumin-positive (PV+) and somatostatin-positive (SST+) cells during adolescence represent changing circuit dynamics that are responsible for the suppression of fear expression between P29 and P35. Further, the sex differences and pubertal influence over PV neuron development may underlie the increased fear of generalization displayed specifically in males (Figure 3 and Figure 4).

Throughout adolescence, inhibitory neurotransmission in mPFC becomes more prominent as inhibitory interneurons develop, assemble into local microcircuits, and refine the dynamics of pyramidal cell activity. After P15, the balance of excitatory to inhibitory inputs onto pyramidal neurons progresses towards greater inhibition (Bouamrane et al., 2016). This coincides with maturation of all the major inhibitory interneuron groups within mPFC including PV, SST, and calretinin (CR) neurons. These changes in inhibition may explain reductions in the basal expression of IEGs between P25 and adulthood (Jia et al., 2018).

Interestingly, PV development differs between the sexes in certain species. In mice, only males show significant increases in PV+ cell density from postnatal weeks 4 to 6 (Du et al., 2018). In rats, both sexes show increased PV+ cell density from P20 to P40 (Holland et al., 2014). Nonetheless, sex is thought to be an important factor in the development of PV neurons in mPFC. Indeed, manipulations that disrupt PV neuron development have sex-specific effects. Reducing the levels of brain-derived neurotrophic factor decreases PV protein expression specifically in males (Du et al., 2018). Female rats exposed to early life stress show decreased PV levels and social deficits earlier than male rats (Holland et al., 2014). Maternal separation also altered the perineuronal nets of PV interneurons in mPFC in a sex-dependent manner (Gildawie et al., 2020). These sex differences are particularly important given that PV+ interneuron numbers (or PV expression) are reduced in neurodevelopmental disorders including ASD, which has a greater preponderance in males (Rapanelli et al., 2017).

These sex-specific findings are also consistent with evidence that pubertal onset and hormone regulation are important influential factors in mPFC interneuron development. Prepubertal gonadectomy in female mice prevents the peri-pubertal increase in inhibitory neurotransmission in the ACC (Piekarski et al., 2017b). Further prepubertal treatment with estradiol and progesterone advanced puberty and inhibitory neurotransmission development (Piekarski et al., 2017b). Together, these studies indicate that sex, pubertal onset, and hormone regulation are important determinants of mPFC development of inhibitory neurotransmission and circuitry.

Despite this, PV expression increases over adolescence in both male and female mice (Du et al., 2018; Caballero et al., 2014). PV interneurons in the cortex are known for their characteristic fast spiking, making them critical modulators of microcircuit function through feed-forward and feed-back inhibition (Hu et al., 2014). PV, a calcium-binding protein, modulates firing rate by regulating intracellular calcium concentrations (Hu et al., 2014). The adolescent increase in PV is consistent with evidence that PV interneurons do not develop their fast-spiking properties in mice until after weaning (Miyamae et al., 2017). If the increase in PV+ expression in adolescence is disrupted, deficits in mPFC gamma-amino butyric acid (GABA)-ergic neurotransmission last into adulthood (Caballero et al., 2020). This defines the upregulation of PV protein in mPFC during adolescence as an important factor that sculpts the local circuit dynamics with lasting effects.

Other classes of interneurons also undergo major developmental changes during adolescence. In contrast to PV, CR, another calcium binding protein that marks a class of non-fast-spiking interneurons, shows decreasing expression throughout adolescence (Du et al., 2018; Caballero et al., 2014). SST protein levels increase over adolescence in females (Du et al., 2018). In males, protein levels stay the same but the number of SST-expressing cells undergoes developmental changes (Du et al., 2018). Together, these findings show that both number of mPFC interneurons expressing PV, SST, or CR and their functional properties change robustly during adolescence albeit with distinct patterns (Figure 1 and Figure 4).

Physiological changes during the development of pyramidal cells are also a critical factor in the maturation of GABAergic transmission. Early in development, intracellular chloride concentration is high such that GABA_A_ receptor activation is initially depolarizing (Rinetti-Vargas et al., 2017). As chloride concentration decreases with age, GABA switches to having a hyperpolarizing effect (Rinetti-Vargas et al., 2017). In prefrontal pyramidal neurons, the time course during which GABA_A_ activation becomes hyperpolarizing differs between the dendrites and the axon initial segment (AIS) (Rinetti-Vargas et al., 2017). In dendrites, the switch to hyperpolarization occurs around P10. In the AIS, however, the timing of this switch is prolonged into adolescence (Rinetti-Vargas et al., 2017). The differential effect of GABA_A_ activation contributes to the distinct developmental profiles of inhibitory circuits as specific classes of GABAergic cells target particular subcellular compartments of pyramidal cells. For example, chandelier cells, a subtype of PV interneurons, synapse specifically onto the AIS where they can control action potential generation and potentially synchronize the output of nearby cells (Somogyi et al., 1982). Thus, different interneuron types are able to mature their functional connections with mPFC pyramidal cells along distinct timelines. Further examination of the distinct trajectories along which inhibitory microcircuits develop is needed.

The differing developmental patterns of these interneuron classes are pertinent in understanding the maturation of mPFC-dependent behaviors. Specific interneuron classes play distinct roles in modulating behavior, particularly learned fear, which is developmentally regulated. For example, phasic inhibition of prefrontal PV interneurons is required for the expression of conditioned fear. Through PV neuron-mediated inhibition, prefrontal projection neurons are disinhibited to facilitate fear expression behavior (Courtin et al., 2014). Plasticity in mPFC SST neurons is essential to encode fear memories, and, through synapses onto PV interneurons, mPFC SST neurons modulate the behavioral expression of conditioned fear (Cummings and Clem, 2020; Figure 3).

The regulatory roles of mPFC interneurons in facilitating fear encoding and expression suggest that the maturation of these neurons is a key event in the developmental regulation of conditioned fear. Interestingly, there is evidence that contextual fear memories are temporarily suppressed between P29 and P35. Mice trained at P29 could not express conditioned fear 24 hr later, but did express conditioned fear 13 days later (Pattwell et al., 2011). This suggests that during early adolescence fear encoding remains intact, but fear expression is inhibited. Given that both PV and SST interneurons within mPFC are necessary for fear expression in the adult (Courtin et al., 2014; Cummings and Clem, 2020), the dynamic changes in protein level, cell morphology, and cell density that occur in these interneuron populations through early adolescence may underlie developmental changes in fear memory retrieval. These changes in interneuron populations are likely to interact with developing long-range connections from key regions including the BLA to influence fear memory retrieval. While strengthening of bidirectional connectivity between mPFC and BLA from P10 to P24 may support the ability of rodents to form persistent fearful memories after weaning, dramatic strengthening of inhibitory transmission in mPFC-BLA circuitry may temporarily disrupt fear memory retrieval in adolescence (Figure 3).

The sex differences observed in PV interneuron development may contribute to sex differences in CFC and fear generalization that emerge during development. Males display greater fear generalization to a novel context following CFC depending on the developmental stage (Colon et al., 2018). This phenotype emerges at P37 for generalization displayed 1 day after training and P33 for generalization displayed 2 weeks after training (Colon et al., 2018). In adults, male rodents have been reported to display greater freezing following CFC than females (Wiltgen et al., 2001; Maren et al., 1994). While this increased freezing has not been reported at adolescent timepoints, linear regression analyses of CFC from P24 to P60 suggest that males display an upward trend of fear expression with age while females display a downward trend (Colon et al., 2018). This suggests that the sex differences observed in adulthood with regards to contextual fear expression may slowly develop through adolescence. These phenotypes may result from the increased PV+ cell density observed throughout adolescence in males but not females (Du et al., 2018).

The development of interneuron populations and inhibitory neurotransmission may also be a key event in the development of cognitively guided behaviors such as reversal learning. In reversal learning tasks, rodents must update their response behaviors when the rules required to receive a food reward are changed. Thus, this behavior relies on persistent motivation for reward, the ability to encode reward memories, and cognitive flexibility. Reversal learning is highly dependent on the orbitofrontal cortex (Groman et al., 2019), and while some reversal tasks have been shown to be insensitive to mPFC inactivation (Floresco et al., 2008; Bissonette et al., 2008), others have resulted in deficits (Kosaki and Watanabe, 2012; Li and Shao, 1998). It has been suggested that mPFC may be important for reversal learning tasks that require greater attentional processes (e.g., difficult discriminanda, visuospatial components) (Bussey et al., 1997; Izquierdo et al., 2017).

The ability of rodents to succeed in reversal learning tasks is developmentally regulated. Juvenile rodents (P26–P27) have been shown to perform well on a four-choice discrimination reversal learning task, learning faster than adults. The performance of both juveniles and adults was reduced following lesions to the dorsomedial frontal cortex (i.e., ACC) (Johnson and Wilbrecht, 2011). The onset of puberty (~P30) appears to disrupt this behavior, leading to worse performance as an adult. Prepubertal hormone treatment used to mimic early pubertal onset reduces the rate reversal learning (Piekarski et al., 2017b). Importantly, mice were able to learn the initial reward association and showed deficits only after the rule switch. Prepubertal hormone treatment was the same manipulation that, as discussed earlier, increased prefrontal inhibitory neurotransmission, linking these two phenomena. This suggests that the onset of puberty and increased inhibitory neurotransmission transiently disrupts the regulation of reward memory to promote adaptive responding. From P30 onward, reversal learning performance steadily increases. While this may be related to refinement of inhibitory circuitry in mPFC, developmental changes in the orbitofrontal cortex are also likely critical to this behavioral change (Moin Afshar et al., 2020). Notably, the rate at which reversal learning was acquired during adolescence predicted performance on the task in adulthood (Moin Afshar et al., 2020). This suggests that developmental processes influencing reversal learning in adolescence may have long-term behavioral effects (Figure 1).

A major persisting gap in our knowledge of the development of inhibitory interneurons and their role in cognitive and emotional behaviors stems from a lack of understanding from a circuit perspective. GABAergic neurons in mPFC receive monosynaptic connections from various long-range inputs; however, when these connections form and how this affects interneuron development is not understood (Sun et al., 2019; Ährlund-Richter et al., 2019). Further, it has been documented that mPFC sends long-range GABAergic projections to subcortical targets but their development also remain uncharacterized (Lee et al., 2014). Given the unique behavioral contributions of different classes of interneurons, mapping out the unique spatiotemporal trajectory of their synaptic development within mPFC microcircuits and performing targeted manipulations of interneuron function during behavior will significantly advance our understanding of how the synaptic development of mPFC inhibitory interneurons shapes maturing behaviors.

## Development of mPFC neuromodulation

The classical neuromodulators dopamine (DA), serotonin (5-hydroxytryptamine [5-HT]), and norepinephrine (NE) are collectively known as monoamine neurotransmitters. These transmitters are released by groups of neurons located in the ventral tegmental area (VTA) and substantia nigra, the raphe nuclei, and the locus coeruleus (LC), respectively. Neurons in these regions send dense projections to the mPFC, where they release neurotransmitter into the synaptic cleft and into the extracellular space outside the synapse. Upon binding to their respective receptors, they can up- or downregulate the membrane potential in target neurons.

In the upcoming sections, we bring together evidence indicating there is an interdependence between the development of the monoamine systems and discuss work relating these developmental processes to the maturation of cognitive function and emotional learning and memory. The 5-HT system develops early and contributes to the structural development of mPFC while providing a necessary foundation for maturation of the DA system. Given the role of mPFC DA in defensive responses, fear learning, and instrumental reward behavior in adulthood, the increase in mPFC DA during adolescence may underlie the maturation of these behaviors. Because the monoamines are linked to many behaviors that are dysregulated in psychiatric disease, the interdependence of their development may explain, in part, the wide variety of phenotypes and genetic mechanisms associated with disease. Thus, mPFC monoaminergic development may present a convergent target for novel therapies for psychiatric illness.

## Maturation of the DA system in mPFC

DA fibers from the VTA heavily innervate the mPFC, where they regulate reward learning, fear conditioning, defensive behaviors, and cognitive control (Reynolds et al., 2018; Vander Weele et al., 2018). DA inputs are already detectable in mPFC at birth and have been observed in L2–L6 by P6 (Kalsbeek et al., 1988). Axonal density of these fibers significantly increases throughout the adolescent period and into early adulthood (Naneix et al., 2012; Willing et al., 2017; Figure 4), with VTA DA axons growing through the nucleus accumbens on their way to mPFC (Reynolds et al., 2018). The magnitude of this innervation is dependent on the disrupted in colorectal cancer (DCC)/Netrin-1 axon guidance system and is vulnerable to disruption through early life drug exposure and psychiatric disease (Reynolds et al., 2018; Manitt et al., 2013; Reynolds et al., 2015). The adolescent maturation of DA axons in mPFC is consistent with the increase in extracellular DA levels in mPFC from P30 to P60 (Niwa et al., 2010). The density of DA receptors increases in the first two postnatal weeks and then declines thereafter ( Leslie et al., 1991; Tarazi and Baldessarini, 2000). This decline stabilizes in early adolescence and is maintained into adulthood (Pokinko et al., 2017; Figure 1). The percentage of DA type 2 receptor (D2R)-expressing neurons within PL neuronal ensembles dramatically increases until the fourth postnatal week and remains constant between P30 and P60 (Yu et al., 2019). Importantly, changes in DA receptor levels throughout adolescence are sexually divergent, with females showing a higher D1R/D2R ratio by early adulthood (Cullity et al., 2019). Early life perturbation, including social defeat and stress (Hill et al., 2014; Watt et al., 2014), is also known to disrupt DA receptor levels.

In adulthood, DA in the mPFC plays a crucial role in regulating responses to aversive stimuli and facilitating fear memory. In mice, DA is released in mPFC during the aversive experience of a tail pinch and biases behavior towards defensive reactions (Vander Weele et al., 2018). This suggests that the development of mPFC DA dynamics may play an integral role in the maturation of defensive behaviors. Throughout adolescence, mPFC becomes increasingly involved in the regulation of innate fear responses. At P14, mPFC is not responsive to innate fear (Chan et al., 2011). By P26, PL is responsive to fearful stimuli but not required for fear behavior (Chan et al., 2011). By mid-adolescence (P38–P42), PL regulates freezing behavior for innate fears (Chan et al., 2011). Importantly, mPFC regulation of innate fear modifies neuronal activity in the ventral periaqueductal gray (PAG). DA release in adult mPFC has been shown to enhance signals in neurons projecting from mPFC to the dorsal PAG (Vander Weele et al., 2018). It remains unclear when this response develops and how it may contribute to the mPFC-PAG activity dynamics in response to aversive stimuli throughout development.

In addition to innate fear, mPFC DA modulates fear learning. D1 and D4 receptors work antagonistically to encode aversive signals during associative fear learning (Lauzon et al., 2009). DA dynamics also play a role in the persistence of fear memories. D1R and D5R activation in mPFC is required for the retention of fear memories over the course of a week (Gonzalez et al., 2014). This is supported by evidence that, depending on the magnitude of DA release in mPFC, it is possible to predict the accuracy of performance on a memory-guided delayed response task (Phillips et al., 2004). At P15, contextual fear memory lasts only 14 hr, but by P30, these fear memories can last at least 28 days (Akers et al., 2012). The adolescent maturation of DA dynamics and receptor densities likely contributes to the emergence of persistent fearful memories during the same period.

mPFC DA also modulates reward-motivated behaviors. While these tasks can be difficult to test in young rodents, it is known that instrumental behavior for food reward changes throughout the adolescent period (Naneix et al., 2012). While adolescent rats learn to lever press for a food reward similarly to adults, they show deficits in altering their behavior following contingency degradation. That is, when an action is no longer required for a reward, adolescent rats continue to perform this action, while adult rats decrease their responding in comparison to another instrumental response required to receive a distinct food reward. However, adolescent rats are still sensitive to reward devaluation and perform Pavlovian to instrumental transfer similarly to adult rats (Naneix et al., 2012; Figure 1). Similar to what has been observed with reversal learning, adolescent rats appear to be able to form associative reward memories but demonstrate impairments in updating these memories.

The maturation of this operant behavior parallels the development of DA axon innervation of mPFC. Importantly, while DA dynamics in the dorsal striatum and nucleus accumbens are also key to reward-learning, innervation by DA axons in these regions does not undergo robust increases during adolescence as occurs in mPFC (Naneix et al., 2012). This poises the development of DA neurotransmission in mPFC as a key event to regulate the maturation of particular aspects of motivated behaviors.

## Maturation of the 5-HT system in mPFC

The role of 5-HT neuromodulation during mPFC development is vast, modulating cellular, circuit, and behavioral development. The 5-HT transporter (5-HTT) directs early morphogenic processes that produce the laminar and cytoarchitectonic structure of mPFC. 5-HTT knockout (KO) mice display increased dendritic branching in mPFC pyramidal cells, decreased number of reelin expressing cells, and changes in morphogen expression that alter cytoarchitectonic development (Wellman et al., 2007; Garcia et al., 2019). The effect of 5-HT release on mPFC pyramidal cells also changes throughout development. Administration of 5-HT to mPFC induces depolarization of L5 pyramidal neurons in pups younger than P19, but shifts to a hyperpolarizing effect commencing during the third postnatal week (Béïque et al., 2004). This progression is due to an age-dependent coordination of depolarizing 5HT7 and 5HT2A receptors and hyperpolarizing 5HT1A receptors. 5HT7 and 5HT1A receptors significantly increase during the second postnatal week and then decrease to adult-like expression levels throughout adolescence (Soiza-Reilly et al., 2019; Goodfellow et al., 2009).

As in the DA system, afferent innervation of mPFC by serotonergic axons is developmentally regulated. A recent study utilizing whole brain mapping of serotonergic axons throughout development in mice observed a distinct temporal innervation pattern of mPFC. In contrast to subcortical targets of serotonergic axons, which displayed gradual increases in innervation throughout the postnatal period, 5-HT-positive axons peaked in mPFC at P7 (Garcia et al., 2019; Maddaloni et al., 2017; Figure 4). Genetic and environmental factors control 5-HT axonal innervation in mPFC. Deleting the cell adhesion protein-encoding gene *Cadherin-13* (*Cdh13*) from embryogenesis positively regulates 5-HT axonal innervation in mPFC (Forero et al., 2017), and early life experiences can modify serotonergic signaling in mPFC (Ohta et al., 2014). As one of the first neuromodulatory systems to develop in mPFC, 5-HT afferents might be uniquely sensitive to the earliest postnatal experiences and are likely to influence a number of subsequent processes during mPFC development. Indeed, the period of peak innervation falls within what has been suggested as a critical period of mPFC 5-HT signaling. Blockade of the 5-HTT between P2 and P11 in mice results in impaired fear extinction in adulthood (Rebello et al., 2014). This manipulation also resulted in dendritic hypertrophy and reduced excitability of pyramidal neurons in IL, the mPFC subregion known to promote extinction (Rebello et al., 2014). Conversely in PL, which promotes fear expression, pyramidal cells became more excitable (Rebello et al., 2014). This exemplifies how early postnatal 5-HT levels modify mPFC cellular properties to influence behavior throughout the lifespan. It is important to note that the critical period of P2–P11 was established in the 129S6/SvEvTac mouse line, which are known to have higher levels of anxiety compared with outbred mice (Rodgers et al., 2002). This may indicate that blockade of 5-HTT converges with other genetic vulnerabilities to produce the behavioral phenotype seen in adulthood.

A study utilizing 5-HTT KO rats found similar evidence that fear extinction in adults was impaired and extended their behavioral investigation to preadolescent (P24) and adolescent (P35) timepoints. Interestingly, they found that while preadolescent rats also displayed impaired fear extinction, adolescent rats showed typical extinction learning (Schipper et al., 2019). This suggests that particular processes occurring in adolescence, perhaps transient states of circuit maturation or synaptic plasticity, temporarily relieve the inhibited fear extinction induced by the absence of 5-HTT. This further illustrates the complex processes that underlie mPFC-dependent behaviors and how the interplay between serotonergic development and other aspects of mPFC development converges to regulate behavioral phenotypes.

**Figure 5.**
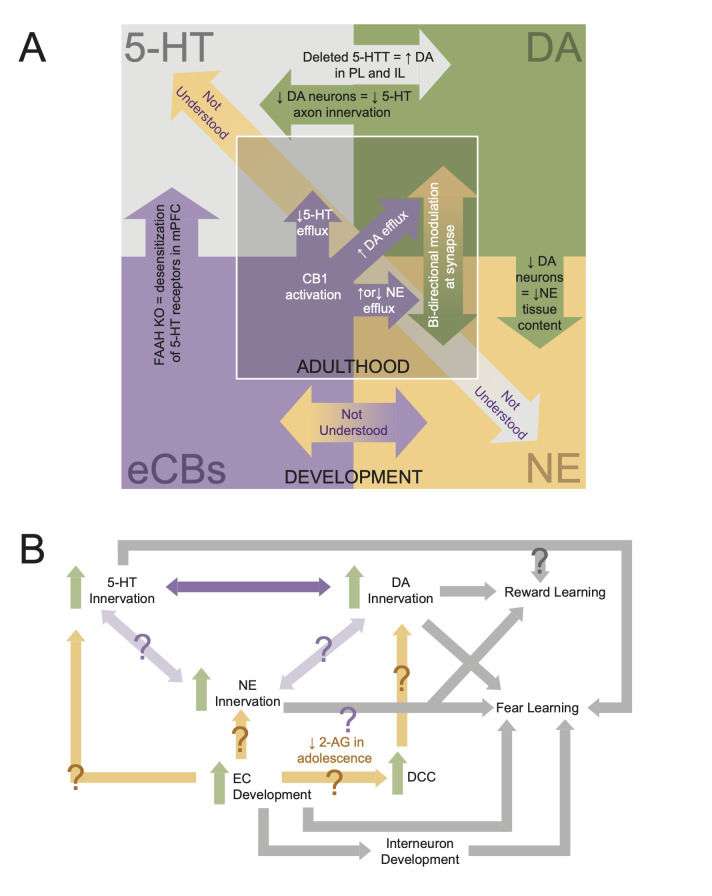
Interdependencies during neuromodulatory system development. (**A**) Schematic showing known interactions between neuromodulatory systems in medial prefrontal cortex (mPFC). Inner square displays phenomena shown in adults, while the outer square displays developmental interactions. (**B**) Flowchart of how development of mPFC neuromodulations converges to give rise to behavior. Arrows with question marks indicate unstudied interactions.

The development of 5-HT and DA systems is highly interconnected. Administration of 6-hydroxydopamine (6-OHDA), which selectively lesions DA neurons, decreases 5-HT innervation of mPFC (Cunningham et al., 2005). Conversely, deleting the 5-HT transporter selectively increases DA innervation in the IL and PL regions of mPFC and decreases DA innervation in the ACC (Garcia et al., 2019; Figure 5). Thus, 5-HT neuromodulation in mPFC plays a critical role in orchestrating mPFC function from early cellular development to shaping neural circuits to guide behavior. Not only can aberrations to the 5-HT system impair fear extinction, as discussed above, but through interaction with DA development, may also affect reward learning and other aspects of fear learning. The mechanisms by which 5-HT and DA development intersect in mPFC require further attention.

## Maturation of the NE system in mPFC

NE axons, originating from the LC, innervate the frontal cortex during embryogenesis and reach adult levels by P6 (Levitt and Moore, 1979). Between P0 and P2, there is a sharp increase in NE tissue content that lowers by P4 and then steadily increases into adulthood (Levitt and Moore, 1979; Figure 4). Like the 5-HT system, there is evidence that the NE system interacts with the DA system in mPFC. Depletion of mPFC DA via local infusion of 6-OHDA from P12–P14 reduced NE content in mPFC tissue in rats aged P30–P65 (Boyce and Finlay, 2009). However, the same study found that DA depletion did not significantly affect NE release in response to a stressful tail pinch (Boyce and Finlay, 2009). Given the prolonged periods of both NE and DA development in mPFC, more studies are needed to determine when and how the DA and NE systems influence one another during mPFC development. Further, our understanding of the interaction between 5-HT and NE in the developing mPFC is entirely lacking (Figure 5). This is despite evidence that these systems are highly intertwined. For example, mice lacking the NE transporter show NE uptake and release by 5-HT terminals in mPFC (Vizi et al., 2004). With the recent development and refinement of intersectional tools for circuit mapping (Fenno, 2020), it is now possible to simultaneously visualize and manipulate different classes of neuromodulatory neurons that project to mPFC within a single brain. These sorts of approaches will be key to determining how interactions between neuromodulatory systems shape mPFC development (Figure 5).

In adults, the release of NE in mPFC plays an important role in the responses to both appetitive and aversive cues. mPFC NE is released in response to both natural and drug reward as well as reward predictive cues (Mingote et al., 2004; Ventura et al., 2008; Ventura et al., 2003; Ventura et al., 2005). The magnitude of NE release is related to the salience of the reward, and thus, NE may serve as a salience signal (Ventura et al., 2008). Consistent with this, NE release in mPFC is required for food-seeking behavior in the presence of potentially harmful consequences (Latagliata et al., 2010). Conditioned release of NE is also observed in response to aversive stimuli and aversive predictive cues (Ventura et al., 2008; Feenstra et al., 1999). This suggests that the development of NE innervation of mPFC may shape the maturation of responses to appetitive and aversive stimuli and that highly salient events during early life may alter the responsiveness of this system. Indeed, unstable maternal care induces prefrontal NE release and results in increased sensitivity to aversive stimuli in adulthood (Ventura et al., 2013). In the future, studies that manipulate NE dynamics in the developing mPFC will be necessary to determine whether NE signaling drives the maturation of reward- or punishment-driven adaptive behaviors.

## Adolescent changes in the mPFC EC system

Fear, stress, and anxiety are modulated by EC signaling within mPFC (Lin et al., 2009; Hill et al., 2011; Fogaça et al., 2012). Impairments in the EC system are observed in a host of psychiatric illnesses including anxiety and depression (Parolaro et al., 2010) . The adolescent period, when many psychiatric illnesses arise, is marked by robust changes in EC signaling. It has been proposed that developmental dynamics in EC signaling may regulate the emergence of fearful and anxious behaviors (Lee et al., 2016). With evidence emerging that EC receptors may play an essential role in circuit development, we outline how the EC system may influence mPFC circuit assembly. We further discuss how genetic mutations associated with the EC system affect mPFC interneuron development, long-range axonal projections, and conditioned fear.

The EC system, which predominantly consists of two inhibitory g-protein coupled receptors (GPCRs; CB1 and CB2) and two ligands (anandamide [AEA] and 2-arachidonoylglycerol [2-AG]), contributes to the balance of excitatory/inhibitory (E/I) synaptic transmission within the mPFC. AEA is a partial agonist of the CB1 receptor while 2-AG is an agonist for both CB1 and CB2 receptors. Serving as a retrograde mechanism to inhibit neurotransmission, EC signaling regulates neuronal excitability.

Adolescence marks a period of dynamic changes in EC signaling within mPFC and other brain regions. In rats, AEA levels in mPFC increase throughout adolescence while levels of 2-AG follow a U-shaped pattern, decreasing from early to mid-adolescence and then increasing from mid- to late adolescence (Ellgren et al., 2008). CB1 receptor expression levels peak around P25, decrease from early to mid-adolescence, and then plateau in late adolescence (Ellgren et al., 2008). Higher expression of CB1 receptors in juveniles coincided with greater CB1-mediated presynaptic inhibition (Heng et al., 2011). Thus, the EC system is likely to be a prime regulator of neuronal excitability prior to the maturation of inhibitory connectivity in mPFC.

Additionally, the EC system stands poised to orchestrate mPFC synapse and circuit development. EC signaling is crucial during the embryonic period for proliferation and specification of pyramidal cell progenitors (Mulder et al., 2008). The CB1 receptor can also act as an axonal guidance cue; however, the role of CB1 in orchestrating mPFC-specific axon guidance is not well understood (Berghuis et al., 2007). Studies conducted in CB1 KO mice have shown that CB1 is required for healthy mPFC development, particularly interneuron development. The loss of CB1 receptors results in decreased levels of the PV protein in PFC (Fitzgerald et al., 2011). Further, altered spatial distribution of D2 receptors and reduced mitochondrial number are observed in PV cells in PFC as a result of CB1 KO (Fitzgerald et al., 2012a). However, because these studies were conducted in mice that lack CB1 throughout their lifetime, we lack an understanding of how ECs alter mPFC function through particular phases of development.

Given the robust changes in concentrations of ECs and their receptors throughout adolescence, it is likely that the adolescent period represents a critical window in which disruption to EC signaling may cause mPFC development to go awry. In support of this, exposure to a CB1 agonist, WIN55,212-2, for a 5-day period during mid (P35–P40) or late (P40–P45) adolescence, resulted in reduced GABAergic transmission in mPFC lasting into adulthood (Cass et al., 2014). Similarly, exposure to tetrahydrocannabinol (THC), the CB1 agonist and psychoactive component of marijuana, during adolescence (P35–P45) caused decreased expression of GAD67, the GABA synthesizing enzyme, and increased pyramidal cell firing in mPFC in adulthood (Renard et al., 2017). Given the necessity of mPFC inhibitory neurotransmission in the expression of conditioned fear, these manipulations may have lifelong behavioral consequences.

EC signaling may also be a key influence over development of the DA system in mPFC. CB1 receptors are colocalized at the same GABAergic terminals as D2 receptors and dopaminergic modulation of EC signaling can result in long-term synaptic depression (Chiu et al., 2010). In retinal cultures, CB1 and CB2 receptors have also been shown to modulate axon guidance through interactions with the DCC/Netrin-1 system (Argaw et al., 2011; Duff et al., 2013). Given that the DCC/Netrin-1 guidance system is critical for development of DA axons in mPFC, if interactions between the EC and DCC/Netrin-1 systems also occur in mPFC, EC activity may influence the extent of dopaminergic innervation. For example, as 2-AG and anandamide levels increase in adolescence, changes in CB1 and CB2 activity may in turn alter DCC expression to promote DA axon innervation and branching. This could support increased and sustained DA innervation of mPFC in adolescence (Figure 5). For a detailed review of the interactions between the EC and DA systems within mPFC, see Fitzgerald et al., 2012b.

The enzyme, fatty acid amide hydrolase (FAAH), regulates levels of AEA, thus modulating CB1 receptor signaling. Mutant mice with reduced FAAH protein levels show increased mPFC axonal innervation of the BLA (Dincheva et al., 2015; Gee et al., 2016). Importantly this phenotype emerges in adolescence as increased axon fiber density is observed in mice at P45 and P75 but not P23 (Gee et al., 2016). Behaviorally, these FAAH-mutated mice show decreased anxiety-like behavior and enhanced fear extinction learning. These findings have been recapitulated in humans with those that express the FAAH A385 allele displaying increased mPFC-amygdala functional connectivity and lower anxiety in adolescence (Dincheva et al., 2015; Gee et al., 2016). Thus, EC signaling regulates structural and functional connectivity of mPFC with important consequences for fear and anxiety-related behaviors.

In adults, EC signaling plays a role in modulating mPFC-dependent behaviors. Levels of ECs modulate conditioned fear. CB1 antagonism increases conditioned freezing while CB2 antagonism decreases this behavior (Llorente-Berzal et al., 2015). Further, inhibiting AEA degradation decreases freezing while inhibiting 2-AG degradation promotes the freezing response (Llorente-Berzal et al., 2015). This suggests that anandamide and 2-AG have opposing influences over the fear response. Importantly, these pharmacological manipulations were given systemically, and it is not known whether mPFC is involved in the effects seen. However, other work has specifically pointed to mPFC ECs in regulating fearful behaviors. mPFC CB1 antagonism disrupts fear extinction (Lin et al., 2009). Inducing hypofunction in N-methyl-D-aspartate receptors (NMDAR) early in life disrupts EC-dependent long-term depression of synapses in mPFC and results in deficits in specifying between fear memories (Lovelace et al., 2014). CB1 receptor activity in BLA neurons that project to mPFC is necessary for associative fear learning (Tan et al., 2010). Thus, EC actions in mPFC and its long-range connections, including with the BLA, contribute to fear learning.

While multiple lines of evidence point to EC signaling as a critical aspect of mPFC development, further work is needed to understand how ECs and their receptors specifically orchestrate mPFC development. CB1 receptor expression changes throughout adolescence and EC signaling are intertwined with monoamine neuromodulatory and inhibitory transmission, which develop along a similar timescale (Figure 1). CB1 receptors likely make important contributions to mPFC pyramidal cell development and axon guidance over long periods of development. Further, the robust changes in concentration of ECs and receptor density over the adolescent period are likely to shape interneuron development and the maturation of fear learning and memory, but causal links are still lacking.

Finally, there is still much to learn about developmental interactions between the EC system and the monoamine neuromodulatory systems in mPFC (Figure 5). Particularly, the developmental interactions between ECs and the 5-HT and NE systems are not well understood. However, it is known that mPFC EC action in adulthood modulates the efflux of 5-HT and NE (Reyes et al., 2012; Morilak, 2012; McLaughlin et al., 2012; Haj-Dahmane and Shen, 2011). For example, a CB1 agonist can either increase or decrease NE efflux in mPFC depending on whether or not a rat was in a state of stress (Reyes et al., 2012). Therefore, the convergence of neuromodulatory development may be a point of vulnerability for experience to disrupt development (Figure 5). Investigating the developmental interactions between neuromodulators is complex. However, by beginning with large-scale manipulations such as receptor and transporter KO lines, and then refining our understanding with cell-type-specific manipulation (e.g., optogenetics or chemogenetics), and receptor manipulation (e.g., pharmacology), we can begin to disentangle these interactions. Notably, understanding how neuromodulatory systems interact in mPFC also requires a more complete developmental understanding of its descending projections to the LC, dorsal raphe, and VTA as modulation of these projection neurons may alter activity in neurons projecting back to mPFC (Figure 2).

## Genetic control of mPFC circuit organization and function

mPFC dysfunction is central to the etiology of neuropsychiatric disorders in which cognitive and emotional behaviors go awry. These include addiction (Hiser and Koenigs, 2018), depression, anxiety (Farrell et al., 2013), ASD, and schizophrenia (Schubert et al., 2015), diseases that all have strong genetic contributions. The prolonged maturation of the developing mPFC permits great regulatory control but also enhances vulnerability to genetic and environmental insults. Up to 50% of these mental conditions emerge by adolescence, with molecular and cellular changes manifesting prior to onset of psychiatric disorders (Shapiro et al., 2017). Studies of cross-species translation in a developmental context have shown that both humans and rodents converge in aspects of mPFC maturation and alignment of critical periods of plasticity (Kolb et al., 2012; Callaghan et al., 2019). Mouse models of schizophrenia and ASD have identified altered expression of signaling molecules and changes in inhibitory circuits that produce abnormal E/I balance and mispositioning of cortical layers and axons in the mPFC (Shapiro et al., 2017; Stoya et al., 2014; Schofield et al., 2011). Taken together, these results provide evidence linking neural signatures of vulnerability to changes in mPFC neurobiology and the behavioral process that rely on those circuits.

Here, we will highlight a series of recent studies that used mouse models to explore the mechanisms of disease progression. We discuss how the genes *Cntnap2, Shank3, Disc1,* and *Dlx5/6* produce overlapping phenotypes in aspects of mPFC synaptic development, which we discussed in previous sections. Specifically, we highlight studies that link developmental changes in inhibitory microcircuit organization and function or long-range synaptic connectivity to cognitive disruptions (Table 1). We also highlight recent work that has identified genetic regulators of DA and 5-HT systems in mPFC. We further postulate about how these developmental circuit disruptions may lead to deficits in regulation of conditioned fear, reward learning, and cognitive flexibility that are observed in neuropsychiatric disorders.

## Genetic regulation of mPFC inhibitory connectivity and emergent circuit functions

Inhibitory neurotransmission shapes the dynamic routing of information through the neocortex (Isaacson and Scanziani, 2011). Growing evidence shows that inhibitory signaling in mPFC undergoes late-stage changes, including dramatic decreases in the E/I balance in mPFC circuits during adolescence. This prolonged period of change likely renders interneurons in mPFC circuits particularly vulnerable to disruption. Indeed, E/I imbalance is considered a pathophysiological mechanism and interneuron dysfunction has been linked to changes in cognition associated with neuropsychiatric diseases that emerge during adolescence, including schizophrenia, impulse control disorders, attention-deficit disorder (ADHD), and ASD (Sohal and Rubenstein, 2019). Elucidating the genetic programs that control the development of mPFC inhibitory interneurons is critical to understand how inhibition shapes the maturation of mPFC-dependent behaviors in health and disease. Here, we discuss several recent studies that reveal convergent roles for *Dlx5/6, Disc1,* and *Cntnap2* in regulating mPFC interneuron physiology and circuit function.

Oscillatory activity can enhance communication between specific brain regions during cognitive tasks. Gamma rhythms driven by PV interneurons are thought to play key roles in cognition and emotional learning (Uhlhaas and Singer, 2010; Cauli et al., 1997; Kawaguchi, 1997; Cardin et al., 2009; Sohal et al., 2009; Fenton et al., 2016). While increased gamma oscillations in PFC have been observed during tasks that require cognitive flexibility, altered gamma oscillations in PFC may be associated with neuropsychiatric disorders including schizophrenia, in which patients exhibit deficits in cognitive flexibility (Green, 2006), impaired fear extinction, and difficulties learning safety cues (Holt et al., 2012). Postmortem analyses have revealed abnormalities in median ganglion eminence (MGE)-derived interneurons, including reduction in the expression of PV and GAD67, a synthetic enzyme for GABA (Volk and Lewis, 2013).

The *Dlx1–6* genes encode a family of homeobox transcription factors that play critical roles in the development of MGE-derived cortical GABAergic interneurons. While *Dlx6* may have limited expression in adult cortical neurons, *Dlx5* is expressed in PV, SST, CR, and neuropeptide Y (NPY) cells in superficial cortical layers, and predominantly in PV cells in deep cortical layers (Wang et al., 2010). *Dlx5/6^-/-^* KOs have reduced numbers of cortical PV cells and increased dendritic branching in the PV cells that remain (Wang et al., 2010). In *Dlx5/6^+/-^* heterozygous mice, alterations in the properties of mPFC PV interneurons arise in early adulthood, beginning around P63. In these mice, PV interneurons have abnormal physiological properties, including wider action potentials, higher input resistance, and slower membrane time constants (Cho et al., 2015a). These changes result in reductions in the amplitude of gamma frequency-induced inhibitory postsynaptic currents in connected mPFC pyramidal neurons. At the same age, *Dlx5/6^+/-^* mice exhibit heightened anxiety and deficits in a rule-shift task that requires cognitive flexibility. Importantly, *Dlx*5/6^+/-^ mice exhibit deficits in task-related gamma frequency power and task performance that can be rescued by pharmacological augmentation of mPFC interneuron function (Cho et al., 2015a). Together, these studies link mutations in *Dlx5* and *Dlx6,* key genes that regulate mPFC interneuron development, to alterations in gamma oscillations that underlie deficits in cognitive flexibility. These mechanisms may contribute to post-adolescent onset of cognitive changes in schizophrenia as well as aberrations in fear and reward learning (Cho et al., 2020).

**Table 1.** Summary of phenotypes in four mouse models. PYR: pyramidal cell; IN: interneuron; MGE: median ganglionic eminence; Pr: release probability; PSD: postsynaptic density; E/I: excitatory/inhibitory.

			Phenotypes		
Gene	Protein function	Cell types	Cellular	Circuit	Behavior
*CNTNAP2*	Axonal transmembrane protein	PYR, INs	Reduced spine density, reduced excitatory and inhibitory synaptic input to PYR cells	Altered phase modulated spiking to delta and theta rhythms, reduced long-range cortico-cortical connectivity, and reduced local connectivity	Repetitive behaviors and cognitive inflexibility
*Disc1*	Intracellular scaffold	PYR, INs, glia	Reduced PV expression, change in Pr in INs, and reduced inhibitory input to PYR cells	Reduced feed-forward inhibition in thalamocortical circuits and elevated E/I ratio	Impairments in working memory, latent inhibition, and pre-pulse inhibition, and increased immobility in forced swim test
*Dlx5/6*	Transcription factor	MGE INs	Deficits in IN migration and reduced IN number	Altered gamma rhythms	Anxiety and congnitive inflexibility
*Shank3*	Excitatory synaptic scaffold	PYR	Reduced dendritic complexity, reduced spine density and PSD length, and reduced excitatory synaptic transmission	Reduced frontostriatal connectivity, reduced local and long-range cortical connectivity, and reduced prefrontal gray matter	Social deficits, anxiety, and repetitive behaviors

Though *Dlx5* and *Dlx6* have not been linked to specific disorders, *Disc1 (disrupted-in schizophrenia-1)* and *Cntnap2* have similar functions in cortical interneurons. DISC1 is a scaffolding protein that interacts with numerous synaptic proteins and enzymes to regulate diverse processes including cortical development and synapse formation (Brandon and Sawa, 2011). A translocation in *DISC1* was reported in a Scottish pedigree as a rare but penetrant risk factor for several mental illnesses including schizophrenia, depression, and bipolar disorder (Millar et al., 2000). Several mouse models of *Disc1* have reductions in PV expression in prefrontal cortex (Niwa et al., 2010; Hikida et al., 2007; Shen et al., 2008; Ibi et al., 2010; Ayhan et al., 2011; Lee et al., 2013) and exhibit impairments in multiple cognitive domains (Niwa et al., 2010; Brandon and Sawa, 2011; Lee et al., 2013; Koike et al., 2006; Clapcote et al., 2007; Li et al., 2007; Kvajo et al., 2008). Until recently, however, the function of *Disc1* in regulating mPFC inhibitory connectivity and circuit function remained unexplored.

Using mice that are heterozygous for the *Disc1* locus impairment (LI) allele, recent work revealed that *Disc1* regulates the connectivity between mPFC pyramidal cells and PV interneurons. Beginning as early as P15, mPFC L2/3 pyramidal cells exhibit reduced inhibitory synaptic input that likely results from reductions in release probability in PV interneurons. These changes have consequences for circuit function, causing a significant decrease in the strength of feed-forward inhibition in the MD thalamus–mPFC pathway (Delevich et al., 2020), one of the most prominent sources of input to the mPFC (DeNardo et al., 2015). The authors also observed elevations in the E/I ratio in mPFC pyramidal neurons (Delevich et al., 2020), a property that is hypothesized to be associated with the pathobiology of neuropsychiatric diseases (Sohal and Rubenstein, 2019; Yizhar et al., 2011). Phenotypic analyses of *Disc1* mutant mice have revealed deficits in cognition (Clapcote et al., 2007). Further, *Disc1* has been shown to interact with cannabis exposure to induce deficits in learned fear, indicating possible vulnerability in the interaction between inhibitory neurotransmission and the EC system (Ballinger et al., 2015). Given the importance of mPFC inhibitory circuit function in these behavioral functions, the identified changes in inhibitory circuits may affect both cognitive and emotive functions.

*Cntnap2* encodes Caspr2, a member of the neurexin family of cell adhesion molecules that is expressed widely throughout the brain in development and in adulthood (Poliak et al., 1999). Mutations in *Cntnap2* are implicated in a human disorder characterized by cognitive and emotional deficits including schizophrenia, obsesive compulsive disorder (OCD), ADHD, and ASD (Strauss et al., 2006; Alarcón et al., 2008; Arking et al., 2008; Bakkaloglu et al., 2008; Peñagarikano et al., 2011). Like *Dlx5/6^+/-^* mice, *Cntnap2* KO mice exhibit changes in the physiological properties of PV cells in addition to reductions in the total number of PV-, NPY-, and CR-positive interneurons (Peñagarikano et al., 2011). PV cells lacking *Cntnap2* have wider spikes, slower membrane time constants, greater adaptation, and more depolarized membrane potentials compared to controls (Vogt et al., 2018). These data suggest that *Cntnap2* may regulate the properties of voltage-dependent sodium and/or potassium channels that mediate action potentials and repolarization in PV interneurons (Vogt et al., 2018).

Consistent with the reduction in the number of cortical interneurons, *Cntnap2^-/-^* L2/3 pyramidal cells in mPFC receive fewer inhibitory synaptic inputs than their wildtype counterparts. As *Cntnap2^-/-^* mice navigate a virtual environment, interneurons exhibit elevated firing rates during both locomotion and immobility. In addition, *Cntnap2* deletion causes reductions in interneuron phase locking to the local field potential (LFP) in delta (4 Hz) and theta (4–8 Hz) frequency ranges and *Cntnap2^-/-^* units tended to fire later in the LFP cycle (Lazaro et al., 2019). Delta oscillations in mPFC have been shown to entrain the amygdala during fear expression (Fujisawa and Buzsáki, 2011), and theta oscillations are associated with signaling safety in fear conditioned animals (Likhtik et al., 2014). Thus, alterations in phase locking of mPFC neurons may give rise to cognitive and affective behavioral dysfunction observed in the *Cntnap2* mouse model (Lazaro et al., 2019).

Based on these studies, PV interneurons and their emergent circuit functions appear to be some of the most vulnerable elements in mPFC circuitry. In several mouse models that exhibit overlapping cognitive deficits, alterations in the physiology and connectivity of mPFC inhibitory interneurons emerge around adolescence. Taken together with work showing that mPFC interneurons undergo major changes in their physiology and circuit functions during adolescence, these studies suggest that the processes that regulate interneuron development during adolescence may be particularly vulnerable to genetic insults. Perturbations to these processes are likely to be determining factors during disease progression. Using floxed alleles and cell-type-specific cre-driver lines, future research can perform spatiotemporally targeted manipulations in mPFC interneurons to refine our understanding of how and when circuit-level deficits contribute to behavioral deficits in cognitive and emotive domains.

## Genetic regulation of excitatory connectivity in mPFC

*Cntnap2* also regulates excitatory connectivity and the physiological properties of mPFC pyramidal cells in a manner that converges with other disease risk genes including *Shank3.* In L5 pyramidal cells, *Cntnap2* deletion causes a reduction in action potential frequency and input resistance specifically in subcortical projection neurons (Brumback et al., 2018). In L2/3, *Cntnap2^-/-^* pyramidal cells exhibit decreases in the strength and number of excitatory synaptic inputs (Lazaro et al., 2019).

In contrast to *Cntnap2,* which is expressed broadly across cell types, the expression of *Shank3* in the PFC is most prevalent in pyramidal cells, with limited expression in GABAergic interneurons, and no apparent expression in glial cells (Guo et al., 2019). *Shank3* encodes an excitatory postsynaptic scaffolding protein that interacts with a variety of postsynaptic density proteins to control dendritic spine morphology and synaptic function (Naisbitt et al., 1999; Sala et al., 2001; Monteiro and Feng, 2017). *Shank3* is also associated with ASD (Durand et al., 2007) as well as with schizophrenia and Phelan–McDermid syndrome, in which patients exhibit ASD-like behaviors including intellectual disability (Phelan and McDermid, 2012). Recent work examined the consequences of *Shank3* deletion in the ACC.

The ACC is implicated in an array of cognitive functions, including decision making, motivation, cost-benefit analyses, and social behaviors (Apps et al., 2016; Chang et al., 2013). *Shank3* KO mice exhibit numerous structural deficits in ACC pyramidal neurons including reductions in dendritic complexity, spine density, and in the length and thickness of postsynaptic densities (Guo et al., 2019). *Shank3* KO neurons also exhibit reductions in both the frequency and amplitude of miniature excitatory postsynaptic currents, consistent with reductions in both the number and strength of excitatory synapses. The decrease in synaptic strength can be attributed to deficits in α-amino-3-hydroxy-5-methyl-4-isoxazole propionic acid receptor-mediated current, while currents through NMDARs were unaffected. Importantly, these deficits can be rescued by targeted delivery of Shank3 to ACC in the adult brain, indicating that Shank3 acts cell autonomously to maintain the strength and number of excitatory synapses in adulthood (Guo et al., 2019).

In addition to regulating local connectivity within prefrontal areas, both *Cntnap2* and *Shank3* KO mice exhibit reductions in prefrontal long-range connectivity. In *Cntnap2^-/-^* mice, decreases in local and long-range functional connectivity are apparent across prefrontal subregions, but these changes are most robust in the cingulate cortex. Regions that exhibit hypoconnectivity with prefrontal areas include the parietal areas, temporal association areas, and hippocampus (Liska et al., 2018). Similarly, *Shank3B^-/-^* homozygous KOs exhibit deficits in local prefrontal connectivity and in long-range connectivity with the retrosplenial (RSP) cortex, ACC, and the striatum. PFC and RSP represent key nodes in the default-mode-network (DMN). In *Shank3B^-/-^* mice, PFC was more disconnected from the DMN. In both mutants, effects were specific to the PFC and motor and sensory networks did not show significant changes in connectivity (Liska et al., 2018).

*Cntnap2* and *Shank3* KO mice exhibit social deficits and motor stereotypies characteristic of ASD and other disorders. However, given their influence over mPFC structural development, synapse formation, and long-range connectivity, both *Cntnap2* and *Shank3* also stand poised to be critical genetic regulators of both fear and reward learning. However, their role in the development of these behaviors is poorly understood. Future investigations may look to these genes as sites of vulnerability in the maturation of cognitive and emotional behaviors controlled by mPFC.

In distinct mouse models, disruptions in inhibitory signaling, synchrony, and long-range connectivity emerge as key points of convergence. These circuit-level signatures may represent important targets for new interventions designed to prevent or ameliorate the symptoms of neuropsychiatric disorders. To date, most studies of genetic mechanisms of mPFC circuit assembly have used whole-animal KOs, in some cases crossing them to transgenic lines that allow them to perform cell-type-specific investigations. These approaches have revealed specific deficits in PV-pyramidal synapses in *Disc1-L1* and *Dlx5/6* mutants. However, given that in these studies the genetic manipulations affected cells across the brain beginning from embryonic development, there are limitations in our understanding of the mechanisms of action and their timing. In the future, studies that use spatially and temporally controlled strategies to manipulate gene expression will provide necessary insights into the cellular and molecular mechanisms underlying the onset of human neuropsychiatric disorders.

## Genetic regulation of mPFC neuromodulatory systems

Dysfunction of the neuromodulatory systems in mPFC is associated with neuropsychiatric diseases in which emotional learning and cognitive flexibility are compromised. Thus, the genes that control the developmental wiring of mPFC neuromodulatory systems may play a critical role in the pathogenesis of such diseases. Dysfunction in mPFC DA signaling has been implicated in schizophrenia (Rao et al., 2019; Howes et al., 2017) and depression (Han and Nestler, 2017). As previously discussed, DA axonal innervation of mPFC dramatically increases throughout the adolescent period and is dependent on DCC/Netrin-1 signaling. *Dcc* knockdown mice show enhanced DA innervation of mPFC and enhanced cognitive flexibility in adulthood (Manitt et al., 2013). This suggests that an increase in DCC expression may cause deficits in cognitive flexibility and contribute to disease phenotypes. Indeed, the rs2270954 polymorphism of the *Dcc* gene was found to be associated with schizophrenia (Grant et al., 2012).

5-HT innervation of mPFC has been associated with *Cdh13*. Mice deficient in *Cdh13* display increased innervation of mPFC compared to control mice (Forero et al., 2017; Forero et al., 2020). Behaviorally, fear extinction is disrupted in male *Cdh13* KO mice but not females. This suggests a sex-dependent mechanism through which *Cdh13* deficiency modulates emotional memory. Importantly, *Cdh13* also directs development in other brain areas such as the hippocampus (Rivero et al., 2015). Thus, further research is needed to understand how the role of *Cdh13* specifically in mPFC may lead to the behavioral impairments seen in *Cdh13* KO mice. This is of great importance given that mutations in *Cdh13* have been associated with a vast number of neuropsychiatric disorders including ADHD (Rivero et al., 2013), depression (Edwards et al., 2012), schizophrenia (Otsuka et al., 2015; Børglum et al., 2014), and bipolar disorder (Cho et al., 2015b).

## Future directions: tackling mPFC complexity in development

The recent circuit analyses in mouse models of neuropsychiatric disorders underscore how mPFC inhibitory microcircuits, neuromodulatory systems, and long-range connectivity – circuit elements that undergo dramatic changes throughout juvenile and adolescent development (Figure 1) – are some of the most vulnerable elements in disease progression. Based on this work, there is an urgent need to refine our understanding of the links between these aspects of mPFC neurodevelopment and maturing behaviors. Uncovering the time course and regulatory mechanisms guiding the assembly of mPFC circuits will refine our understanding of which developmental milestones have profound effects on mPFC function and how they come about. This knowledge will be key to understanding the neural basis of behavioral transitions during development and can inform targeted manipulations of mPFC circuit elements to directly test their behavioral functions at distinct developmental stages. For instance, during the juvenile period, new work can test whether the strengthening of long-range mPFC connections with regions including the BLA contributes to mPFC’s increasing role in fear and reward learning and a developmental switch from short- to long-lasting fear memories (Figure 1 and Figure 3). In adolescence, targeted manipulations of inhibitory and neuromodulatory cell types can test whether strengthening synaptic inhibition and refinement of mPFC neuromodulation are required for the maturation of reward learning, conditioned fear, and cognitive flexibility (Figure 1 and Figure 4). Establishing links between neurodevelopment and behavior is an essential first step to understanding how and when early insults transform these processes and can inform new interventional strategies to slow or prevent disease progression.

With the explosion of viral and genetic approaches over the last 10 years, we have tools in hand to manipulate these developmental processes with unprecedented specificity and resolution (Luo et al., 2018). Thereby, we can directly link the maturation of cells and circuits to the maturation of complex behaviors. The emergent hypotheses we put forth in this review can be tested using viral-genetic approaches to (1) define causal links between specific aspects of cellular and circuit development and cognitive and emotive functions, (2) understand how the developmental processes we describe are contingent on one another, and (3) discover the molecular and cellular programs that control the maturation of mPFC connectivity. Genes that wire specific mPFC synaptic connections can form the basis of new genetic tools to target developing circuit elements with increasing precision.

Developmental changes in the expression of emotional memories and cognitive flexibility may rely on developmental milestones within mPFC inhibitory microcircuits. Around the same time that mPFC inhibitory interneurons undergo changes in the expression of key signaling proteins (e.g., PV and SST) and mPFC inhibitory synaptic currents strengthen dramatically, animals exhibit temporary deficits in fear memory retrieval, increases in fear memory generalization, decreased performance in reversal learning, and insensitivity to reward contingency degradation. Thereafter, as inhibitory circuitry is refined, fear memory becomes robust and persistent, reward learning becomes sensitive to contingency degradation, and performance improves in reversal learning tasks (Figure 1). This suggests that precise inhibitory control over the timing or levels of mPFC activity is necessary for mature forms of cognitive and emotive behavior to emerge. It is unclear whether connections between mPFC interneurons and distinct classes of pyramidal cells uniquely control the maturation of these different behavioral functions, or whether there is a common mechanism governing the observed changes in each behavioral domain. Determining whether this is the case will require a more detailed understanding of synaptic development between classes of mPFC interneurons and pyramidal cells and new research that performs targeted manipulations of activity, connectivity, and synaptic function in different mPFC neuronal types during behavior in developing animals.

Conceptually similar approaches can also be applied to determine how the developing DA, 5-HT, and NE systems shape the maturation of emotional memory and cognitive flexibility. For instance, increased innervation of DA axons in mPFC may promote the maturation of defensive behaviors (i.e., freezing), the retention of fear memory, and instrumental learning, while 5-HT signaling in adolescence may be critical for the development of fear extinction. The development of NE signaling dynamics may promote the attribution of salience and learned associations to reward and fear cues. Addressing these questions will require a more detailed understanding of how neuromodulation shapes both behavior and the underlying neural signaling at different stages of development. We can leverage transgenic tools for activity dependent genetic labeling (DeNardo and Luo, 2017) to capture behaviorally activated neurons in the developing brain and then assess their behavioral functions at later times using invasive methods such as Ca^2+^ imaging. By performing developmental activity-based tagging with concurrent manipulations of neuromodulatory signaling, we may be able to determine how neuromodulation shapes the neural basis of behavior at juvenile and adolescent stages.

Proper assembly of mPFC circuits requires temporally precise orchestration of multiple interdependent systems. From birth through adolescence, the DA, NE, 5-HT, and EC systems influence each other’s development in ways we are only beginning to understand (Figure 5). As the 5-HT system is one of the earliest to form connections, it might have particularly strong influence over the maturation of the other monoamine systems as they innervate mPFC. Global manipulations of 5-HT signaling during specific developmental windows cause concurrent changes in DA levels in mPFC and impair fear extinction in adulthood. To test whether these interactions occur locally within mPFC, new research could combine viral tracing with genetic deletion of the 5-HT synthetic enzyme tryptophan hydroxylase 2, *Tph2*. By disrupting 5-HT signaling specifically in projections to mPFC during development, new research can ask whether DA signaling is reduced in a corresponding way. Using intersectional genetic strategies to simultaneously visualize 5-HT and DA axons in mPFC within a single brain can further reveal how disruptions in 5-HT signaling impact the development of both systems. As EC signaling has been shown to regulate DA receptors levels and to interact with Dcc/Netrin-1 signaling to promote axon guidance in the retina, it may regulate development of DA axons in mPFC. The neuromodulatory systems are exquisitely sensitive to early occurring environmental changes and genetic mutations that have lasting behavioral consequences. Because of this, understanding how their development is intertwined may help to explain the spectrum of behavioral phenotypes associated with neuropsychiatric disorders.

Precise wiring of mPFC long-range connections is essential for mPFC function in adulthood, but our understanding of the timeline along which specific pathways mature and how circuit maturation shapes behavior is lacking. In the case of fear learning and memory, mPFC circuits known to play a role in the adult brain may also underlie the developmental regulation of fearful memories. Bidirectional connectivity between mPFC and BLA increases dramatically during the fourth postnatal week. At the same time, fear memories become robust and long-lasting. As such, the developmental strengthening of mPFC-BLA connectivity may be essential to establish prefrontal control of the expression of conditioned fear. To test whether these processes are causally linked, new research can determine whether increasing activity in the mPFC-BLA pathway during fear learning and memory in juvenile development enhances the ability to form long-lasting memories. Interestingly, reward learning and cognitive flexibility mature along a similar timeline (Figure 1) and deficits in long-range connectivity are hallmarks of psychiatric disease (Table 1). Thus, enhanced regulatory control that comes with the maturation of mPFC long-range synaptic connectivity may have broad affects, allowing mature forms of numerous cognitive and emotional behaviors to emerge.

Importantly, the development of many mPFC connections that play key roles in the regulation of emotional memory and cognition, including connections with the medial entorhinal cortex and midline thalamic nuclei, has not been carefully examined in development (Figure 2). In the case of conditioned fear, research that explores the anatomical and functional maturation of these connections, both in naive animals and in those that have undergone fear conditioning in juvenile stages when fear memories are short-lived, can link developmental changes in synaptic function to age-dependent differences in fear memory robustness. Arruda-Carvalho et al. provide an elegant example of how to describe the structural and functional maturation of an mPFC pathway using channelrhodopsin (ChR2)-assisted circuit mapping (Arruda-Carvalho et al., 2017). With this approach, in which mPFC axons are transduced with ChR2 and light-evoked postsynaptic currents are recorded in target cells in a downstream region of interest, we can understand precisely when long-range mPFC synaptic connections form and functionally mature, and how they respond to learning. Given that individual mPFC pathways have been linked to distinct behavioral functions, synaptic connections that mature along different developmental timelines may regulate maturing behaviors during distinct developmental windows. This information will be important for understanding windows of vulnerability during disease progression.

As we gain a more detailed understanding of when particular mPFC connections form and mature, we can begin to investigate the underlying molecular mechanisms. Extracellular signals can be important modulators of synapse development. For instance, the innervation of BLA by mPFC axons is modulated by levels of ECs. As discussed, manipulations to the EC system only affect mPFC→BLA neurons at the beginning of adolescence, suggesting a specific temporal window in which EC signaling may direct mPFC circuit maturation. To hone-in on the precise temporal dynamics with which the EC system acts on mPFC circuitry, conditional KOs or pharmacological manipulations of EC signaling can be performed at discrete time points throughout the developmental period. Further, the EC system may modulate the extent of innervation in other mPFC target regions. Future studies can look at how manipulations in EC levels affect the extent of axon innervation in other mPFC target regions important for fear and reward learning and cognition, including thalamic nuclei and striatal regions.

In addition to external influences from neuromodulatory signals, genes encoding synaptic organizer proteins may also play a critical role in wiring mPFC connections. For instance, *Cdh8* is expressed selectively in mPFC neurons that project to the striatum. Given its role in target selection and synaptic plasticity in the retina and hippocampus, *Cdh8* may specifically regulate wiring of mPFC-striatal synapses. *Cdh13* regulates the development of 5-HT axons in mPFC, and the guidance cues Dcc/Netrin-1 regulate wiring of DA fibers in mPFC, though it remains unclear whether Cdh13 and Dcc/Netrin-1 exclusively regulate 5-HT and DA axons in mPFC, or if their function extends to other pathways as well. If *Cdh8*, *Cdh13*, and *Dcc/Netrin-1* indeed play specific roles in wiring frontostriatal, serotonergic, and dopaminergic connectivity, respectively, we can then leverage them as tools to manipulate those pathways and measure the impact on developing behaviors. As *Cdh8*, *Cdh13,* and *Dcc/Netrin-1* have been linked to ASD, depression, and schizophrenia, investigating their role in wiring the healthy brain can provide important clues about how mPFC circuitry is perturbed in disease.

Major hurdles in developmental circuit mapping stem from the challenge of precise stereotaxic targeting in small developing brains. Now, researchers often rely on brute-force approaches to target specific regions in early postnatal development. Wiring specificity genes like *Cdh8*, whose differential expression patterns in the nervous system allow them to regulate the formation and maturation of specific classes of synapses, could eventually form the basis of new cre-driver lines. New transgenic lines that provide genetic access to particular classes of developing mPFC neurons can ease our reliance on precise stereotaxic targeting in early postnatal stages. In combination with floxed alleles, optogenetics, chemogenetics, and genetically encoded Ca^2+^ indicators, new cre-driver lines that provide access to particular developing circuit elements will allow us to link the assembly of mPFC circuits to maturing behaviors with unprecedented specificity.

## References

[bib1] Adriani W, Granstrem O, Macri S, Izykenova G, Dambinova S, Laviola G (2004). Behavioral and neurochemical vulnerability during adolescence in mice: studies with nicotine. Neuropsychopharmacology.

[bib2] Agoglia AE, Holstein SE, Small AT, Spanos M, Burrus BM, Hodge CW (2017). Comparison of the adolescent and adult mouse prefrontal cortex proteome. PLOS ONE.

[bib3] Ährlund-Richter S, Xuan Y, van Lunteren JA, Kim H, Ortiz C, Pollak Dorocic I, Meletis K, Carlén M (2019). A whole-brain atlas of monosynaptic input targeting four different cell types in the medial prefrontal cortex of the mouse. Nature Neuroscience.

[bib4] Akers KG, Arruda-Carvalho M, Josselyn SA, Frankland PW (2012). Ontogeny of contextual fear memory formation, specificity, and persistence in mice. Learning & Memory.

[bib5] Akers KG, Martinez-Canabal A, Restivo L, Yiu AP, De Cristofaro A, Hsiang H-L, Wheeler AL, Guskjolen A, Niibori Y, Shoji H, Ohira K, Richards BA, Miyakawa T, Josselyn SA, Frankland PW (2014). Hippocampal neurogenesis regulates forgetting during adulthood and infancy. Science.

[bib6] Alarcón M, Abrahams BS, Stone JL, Duvall JA, Perederiy JV, Bomar JM, Sebat J, Wigler M, Martin CL, Ledbetter DH, Nelson SF, Cantor RM, Geschwind DH (2008). Linkage, association, and gene-expression analyses identify CNTNAP2 as an autism-susceptibility gene. The American Journal of Human Genetics.

[bib7] Apps MA, Rushworth MF, Chang SW (2016). The anterior cingulate gyrus and social cognition: tracking the motivation of others. Neuron.

[bib8] Argaw A, Duff G, Zabouri N, Cécyre B, Chainé N, Cherif H, Tea N, Lutz B, Ptito M, Bouchard JF (2011). Concerted action of CB1 cannabinoid receptor and deleted in colorectal Cancer in axon guidance. Journal of Neuroscience.

[bib9] Arking DE, Cutler DJ, Brune CW, Teslovich TM, West K, Ikeda M, Rea A, Guy M, Lin S, Cook EH, Chakravarti A (2008). A common genetic variant in the neurexin superfamily member CNTNAP2 increases familial risk of autism. The American Journal of Human Genetics.

[bib10] Arruda-Carvalho M, Wu WC, Cummings KA, Clem RL (2017). Optogenetic examination of Prefrontal-Amygdala synaptic development. The Journal of Neuroscience.

[bib11] Ayhan Y, Abazyan B, Nomura J, Kim R, Ladenheim B, Krasnova IN, Sawa A, Margolis RL, Cadet JL, Mori S, Vogel MW, Ross CA, Pletnikov MV (2011). Differential effects of prenatal and postnatal expressions of mutant human DISC1 on neurobehavioral phenotypes in transgenic mice: evidence for neurodevelopmental origin of major psychiatric disorders. Molecular Psychiatry.

[bib12] Backus AR, Schoffelen JM, Szebényi S, Hanslmayr S, Doeller CF (2016). Hippocampal-Prefrontal theta oscillations support memory integration. Current Biology.

[bib13] Bakkaloglu B, O'Roak BJ, Louvi A, Gupta AR, Abelson JF, Morgan TM, Chawarska K, Klin A, Ercan-Sencicek AG, Stillman AA, Tanriover G, Abrahams BS, Duvall JA, Robbins EM, Geschwind DH, Biederer T, Gunel M, Lifton RP, State MW (2008). Molecular cytogenetic analysis and resequencing of contactin associated protein-like 2 in autism spectrum disorders. The American Journal of Human Genetics.

[bib14] Ballinger MD, Saito A, Abazyan B, Taniguchi Y, Huang CH, Ito K, Zhu X, Segal H, Jaaro-Peled H, Sawa A, Mackie K, Pletnikov MV, Kamiya A (2015). Adolescent Cannabis exposure interacts with mutant DISC1 to produce impaired adult emotional memory. Neurobiology of Disease.

[bib15] Béïque JC, Campbell B, Perring P, Hamblin MW, Walker P, Mladenovic L, Andrade R (2004). Serotonergic regulation of membrane potential in developing rat prefrontal cortex: coordinated expression of 5-hydroxytryptamine (5-HT)1A, 5-HT2A, and 5-HT7 receptors. Journal of Neuroscience.

[bib16] Bell MR (2018). Comparing postnatal development of gonadal hormones and associated social behaviors in rats, mice, and humans. Endocrinology.

[bib17] Berghuis P, Rajnicek AM, Morozov YM, Ross RA, Mulder J, Urbán GM, Monory K, Marsicano G, Matteoli M, Canty A, Irving AJ, Katona I, Yanagawa Y, Rakic P, Lutz B, Mackie K, Harkany T (2007). Hardwiring the brain: endocannabinoids shape neuronal connectivity. Science.

[bib18] Bissonette GB, Martins GJ, Franz TM, Harper ES, Schoenbaum G, Powell EM (2008). Double dissociation of the effects of medial and orbital prefrontal cortical lesions on attentional and affective shifts in mice. Journal of Neuroscience.

[bib19] Børglum AD, Demontis D, Grove J, Pallesen J, Hollegaard MV, Pedersen CB, Hedemand A, Mattheisen M, Uitterlinden A, Nyegaard M, Ørntoft T, Wiuf C, Didriksen M, Nordentoft M, Nöthen MM, Rietschel M, Ophoff RA, Cichon S, Yolken RH, Hougaard DM, Mortensen PB, Mors O, GROUP investigators10 (2014). Genome-wide study of association and interaction with maternal Cytomegalovirus infection suggests new schizophrenia loci. Molecular Psychiatry.

[bib20] Bouamrane L, Scheyer AF, Lassalle O, Iafrati J, Thomazeau A, Chavis P (2016). Reelin-Haploinsufficiency disrupts the developmental trajectory of the E/I balance in the prefrontal cortex. Frontiers in Cellular Neuroscience.

[bib21] Boyce PJ, Finlay JM (2009). Extracellular dopamine and norepinephrine in the developing rat prefrontal cortex: transient effects of early partial loss of dopamine. Brain Research Bulletin.

[bib22] Brandon NJ, Sawa A (2011). Linking neurodevelopmental and synaptic theories of mental illness through DISC1. Nature Reviews Neuroscience.

[bib23] Brockmann MD, Pöschel B, Cichon N, Hanganu-Opatz IL (2011). Coupled oscillations mediate directed interactions between prefrontal cortex and Hippocampus of the neonatal rat. Neuron.

[bib24] Brumback AC, Ellwood IT, Kjaerby C, Iafrati J, Robinson S, Lee AT, Patel T, Nagaraj S, Davatolhagh F, Sohal VS (2018). Identifying specific prefrontal neurons that contribute to autism-associated abnormalities in physiology and social behavior. Molecular Psychiatry.

[bib25] Brust V, Schindler PM, Lewejohann L (2015). Lifetime development of behavioural phenotype in the house mouse *Mus musculus*. Frontiers in Zoology.

[bib26] Bussey TJ, Muir JL, Everitt BJ, Robbins TW (1997). Triple dissociation of anterior Cingulate, posterior Cingulate, and medial frontal cortices on visual discrimination tasks using a touchscreen testing procedure for the rat. Behavioral Neuroscience.

[bib27] Caballero A, Flores-Barrera E, Cass DK, Tseng KY (2014). Differential regulation of parvalbumin and calretinin interneurons in the prefrontal cortex during adolescence. Brain Structure and Function.

[bib28] Caballero A, Flores-Barrera E, Thomases DR, Tseng KY (2020). Downregulation of parvalbumin expression in the prefrontal cortex during adolescence causes enduring prefrontal disinhibition in adulthood. Neuropsychopharmacology.

[bib29] Callaghan B, Meyer H, Opendak M, Van Tieghem M, Harmon C, Li A, Lee FS, Sullivan RM, Tottenham N (2019). Using a developmental ecology framework to align fear neurobiology across species. Annual Review of Clinical Psychology.

[bib30] Cardin JA, Carlén M, Meletis K, Knoblich U, Zhang F, Deisseroth K, Tsai LH, Moore CI (2009). Driving fast-spiking cells induces gamma rhythm and controls sensory responses. Nature.

[bib31] Cass DK, Flores-Barrera E, Thomases DR, Vital WF, Caballero A, Tseng KY (2014). CB1 cannabinoid receptor stimulation during adolescence impairs the maturation of GABA function in the adult rat prefrontal cortex. Molecular Psychiatry.

[bib32] Cauli B, Audinat E, Lambolez B, Angulo MC, Ropert N, Tsuzuki K, Hestrin S, Rossier J (1997). Molecular and physiological diversity of cortical nonpyramidal cells. The Journal of Neuroscience.

[bib33] Chan T, Kyere K, Davis BR, Shemyakin A, Kabitzke PA, Shair HN, Barr GA, Wiedenmayer CP (2011). The role of the medial prefrontal cortex in innate fear regulation in infants, juveniles, and adolescents. Journal of Neuroscience.

[bib34] Chang SW, Gariépy JF, Platt ML (2013). Neuronal reference frames for social decisions in primate frontal cortex. Nature Neuroscience.

[bib35] Chiu CQ, Puente N, Grandes P, Castillo PE (2010). Dopaminergic modulation of endocannabinoid-mediated plasticity at GABAergic synapses in the prefrontal cortex. Journal of Neuroscience.

[bib36] Cho KK, Hoch R, Lee AT, Patel T, Rubenstein JL, Sohal VS (2015a). Gamma rhythms link prefrontal interneuron dysfunction with cognitive inflexibility in dlx5/6+/- mice. Neuron.

[bib37] Cho CH, Lee HJ, Woo HG, Choi JH, Greenwood TA, Kelsoe JR (2015b). *CDH13* and *HCRTR2* may be associated with hypersomnia symptom of bipolar depression: a Genome-Wide functional enrichment pathway analysis. Psychiatry Investigation.

[bib38] Cho KKA, Davidson TJ, Bouvier G, Marshall JD, Schnitzer MJ, Sohal VS (2020). Cross-hemispheric gamma synchrony between prefrontal parvalbumin interneurons supports behavioral adaptation during rule shift learning. Nature Neuroscience.

[bib39] Clapcote SJ, Lipina TV, Millar JK, Mackie S, Christie S, Ogawa F, Lerch JP, Trimble K, Uchiyama M, Sakuraba Y, Kaneda H, Shiroishi T, Houslay MD, Henkelman RM, Sled JG, Gondo Y, Porteous DJ, Roder JC (2007). Behavioral phenotypes of Disc1 missense mutations in mice. Neuron.

[bib40] Colon L, Odynocki N, Santarelli A, Poulos AM (2018). Sexual differentiation of contextual fear responses. Learning & Memory.

[bib41] Courtin J, Chaudun F, Rozeske RR, Karalis N, Gonzalez-Campo C, Wurtz H, Abdi A, Baufreton J, Bienvenu TC, Herry C (2014). Prefrontal parvalbumin interneurons shape neuronal activity to drive fear expression. Nature.

[bib42] Cullity ER, Madsen HB, Perry CJ, Kim JH (2019). Postnatal developmental trajectory of dopamine receptor 1 and 2 expression in cortical and striatal brain regions. Journal of Comparative Neurology.

[bib43] Cummings KA, Clem RL (2020). Prefrontal somatostatin interneurons encode fear memory. Nature Neuroscience.

[bib44] Cunningham MG, Bhattacharyya S, Benes FM (2002). Amygdalo-cortical sprouting continues into early adulthood: implications for the development of normal and abnormal function during adolescence. The Journal of Comparative Neurology.

[bib45] Cunningham MG, Connor CM, Zhang K, Benes FM (2005). Diminished serotonergic innervation of adult medial prefrontal cortex after 6-OHDA lesions in the newborn rat. Developmental Brain Research.

[bib46] Delevich K, Thomas AW, Wilbrecht L (2018). Adolescence and 'late blooming' synapses of the prefrontal cortex.

[bib47] Delevich K, Jaaro-Peled H, Penzo M, Sawa A, Li B (2020). Parvalbumin interneuron dysfunction in a Thalamo-Prefrontal cortical circuit in *Disc1* Locus Impairment Mice. Eneuro.

[bib48] DeNardo LA, Berns DS, DeLoach K, Luo L (2015). Connectivity of mouse somatosensory and prefrontal cortex examined with trans-synaptic tracing. Nature Neuroscience.

[bib49] DeNardo L, Luo L (2017). Genetic strategies to access activated neurons. Current Opinion in Neurobiology.

[bib50] Dincheva I, Drysdale AT, Hartley CA, Johnson DC, Jing D, King EC, Ra S, Gray JM, Yang R, DeGruccio AM, Huang C, Cravatt BF, Glatt CE, Hill MN, Casey BJ, Lee FS (2015). FAAH genetic variation enhances fronto-amygdala function in mouse and human. Nature Communications.

[bib51] Du X, Serena K, Hwang WJ, Grech AM, Wu YWC, Schroeder A, Hill RA (2018). Prefrontal cortical parvalbumin and somatostatin expression and cell density increase during adolescence and are modified by BDNF and sex. Molecular and Cellular Neuroscience.

[bib52] Duff G, Argaw A, Cecyre B, Cherif H, Tea N, Zabouri N, Casanova C, Ptito M, Bouchard JF (2013). Cannabinoid receptor CB2 modulates axon guidance. PLOS ONE.

[bib53] Durand CM, Betancur C, Boeckers TM, Bockmann J, Chaste P, Fauchereau F, Nygren G, Rastam M, Gillberg IC, Anckarsäter H, Sponheim E, Goubran-Botros H, Delorme R, Chabane N, Mouren-Simeoni MC, de Mas P, Bieth E, Rogé B, Héron D, Burglen L, Gillberg C, Leboyer M, Bourgeron T (2007). Mutations in the gene encoding the synaptic scaffolding protein SHANK3 are associated with autism spectrum disorders. Nature Genetics.

[bib54] Edwards AC, Aliev F, Bierut LJ, Bucholz KK, Edenberg H, Hesselbrock V, Kramer J, Kuperman S, Nurnberger JI, Schuckit MA, Porjesz B, Dick DM (2012). Genome-wide association study of comorbid depressive syndrome and alcohol dependence. Psychiatric Genetics.

[bib55] Ellgren M, Artmann A, Tkalych O, Gupta A, Hansen HS, Hansen SH, Devi LA, Hurd YL (2008). Dynamic changes of the endogenous cannabinoid and opioid mesocorticolimbic systems during adolescence: thc effects. European Neuropsychopharmacology.

[bib56] Farrell MR, Sengelaub DR, Wellman CL (2013). Sex differences and chronic stress effects on the neural circuitry underlying fear conditioning and extinction. Physiology & Behavior.

[bib57] Feenstra MG, Teske G, Botterblom MH, De Bruin JP (1999). Dopamine and noradrenaline release in the prefrontal cortex of rats during classical aversive and appetitive conditioning to a contextual stimulus: interference by novelty effects. Neuroscience Letters.

[bib58] Fenno LE (2020). Comprehensive dual- and Triple-Feature intersectional Single-Vector delivery of diverse functional payloads to cells of behaving mammals. Journal of Cleaner Production.

[bib59] Fenton GE, Halliday DM, Mason R, Bredy TW, Stevenson CW (2016). Sex differences in learned fear expression and extinction involve altered gamma oscillations in medial prefrontal cortex. Neurobiology of Learning and Memory.

[bib60] Ferguson BR, Gao WJ (2014). Development of thalamocortical connections between the mediodorsal thalamus and the prefrontal cortex and its implication in cognition. Frontiers in Human Neuroscience.

[bib61] Fitzgerald ML, Lupica CR, Pickel VM (2011). Decreased parvalbumin immunoreactivity in the cortex and striatum of mice lacking the CB1 receptor. Synapse.

[bib62] Fitzgerald ML, Chan J, Mackie K, Lupica CR, Pickel VM (2012a). Altered dendritic distribution of dopamine D2 receptors and reduction in mitochondrial number in parvalbumin-containing interneurons in the medial prefrontal cortex of cannabinoid-1 (CB1) receptor knockout mice. Journal of Comparative Neurology.

[bib63] Fitzgerald ML, Shobin E, Pickel VM (2012b). Cannabinoid modulation of the dopaminergic circuitry: implications for limbic and striatal output. Progress in Neuro-Psychopharmacology and Biological Psychiatry.

[bib64] Floresco SB, Block AE, Tse MT (2008). Inactivation of the medial prefrontal cortex of the rat impairs strategy set-shifting, but not reversal learning, using a novel, automated procedure. Behavioural Brain Research.

[bib65] Fogaça MV, Aguiar DC, Moreira FA, Guimarães FS (2012). The endocannabinoid and endovanilloid systems interact in the rat prelimbic medial prefrontal cortex to control anxiety-like behavior. Neuropharmacology.

[bib66] Forero A, Rivero O, Wäldchen S, Ku HP, Kiser DP, Gärtner Y, Pennington LS, Waider J, Gaspar P, Jansch C, Edenhofer F, Resink TJ, Blum R, Sauer M, Lesch KP (2017). Cadherin-13 deficiency increases dorsal raphe 5-HT neuron density and prefrontal cortex innervation in the mouse brain. Frontiers in Cellular Neuroscience.

[bib67] Forero A, Ku HP, Malpartida AB, Wäldchen S, Alhama-Riba J, Kulka C, Aboagye B, Norton WHJ, Young AMJ, Ding YQ, Blum R, Sauer M, Rivero O, Lesch KP (2020). Serotonin (5-HT) neuron-specific inactivation of Cadherin-13 impacts 5-HT system formation and cognitive function. Neuropharmacology.

[bib68] Fujisawa S, Buzsáki G (2011). A 4 hz oscillation adaptively synchronizes prefrontal, VTA, and hippocampal activities. Neuron.

[bib69] Gabbott PLA, Warner TA, Jays PRL, Salway P, Busby SJ (2005). Prefrontal cortex in the rat: Projections to Subcortical Autonomic, Motor, and Limbic Centers. The Journal of Comparative Neurology.

[bib70] Garcia LP, Witteveen JS, Middelman A, van Hulten JA, Martens GJM, Homberg JR, Kolk SM (2019). Perturbed developmental serotonin signaling affects prefrontal catecholaminergic innervation and cortical integrity. Molecular Neurobiology.

[bib71] Gee DG, Fetcho RN, Jing D, Li A, Glatt CE, Drysdale AT, Cohen AO, Dellarco DV, Yang RR, Dale AM, Jernigan TL, Lee FS, Casey BJ, PING Consortium (2016). Individual differences in frontolimbic circuitry and anxiety emerge with adolescent changes in endocannabinoid signaling across species. PNAS.

[bib72] Gildawie KR, Honeycutt JA, Brenhouse HC (2020). Region-specific effects of maternal separation on perineuronal net and Parvalbumin-expressing interneuron formation in male and female rats. Neuroscience.

[bib73] Gonzalez MC, Kramar CP, Tomaiuolo M, Katche C, Weisstaub N, Cammarota M, Medina JH (2014). Medial prefrontal cortex dopamine controls the persistent storage of aversive memories. Frontiers in Behavioral Neuroscience.

[bib74] Goodfellow NM, Benekareddy M, Vaidya VA, Lambe EK (2009). Layer II/III of the prefrontal cortex: inhibition by the serotonin 5-HT1A receptor in development and stress. Journal of Neuroscience.

[bib75] Grant A, Fathalli F, Rouleau G, Joober R, Flores C (2012). Association between schizophrenia and genetic variation in DCC: a case-control study. Schizophrenia Research.

[bib76] Green MF (2006). Cognitive impairment and functional outcome in schizophrenia and bipolar disorder. The Journal of Clinical Psychiatry.

[bib77] Groman SM, Keistler C, Keip AJ, Hammarlund E, DiLeone RJ, Pittenger C, Lee D, Taylor JR (2019). Orbitofrontal circuits control multiple Reinforcement-Learning processes. Neuron.

[bib78] Guo B, Chen J, Chen Q, Ren K, Feng D, Mao H, Yao H, Yang J, Liu H, Liu Y, Jia F, Qi C, Lynn-Jones T, Hu H, Fu Z, Feng G, Wang W, Wu S (2019). Anterior cingulate cortex dysfunction underlies social deficits in Shank3 mutant mice. Nature Neuroscience.

[bib79] Haj-Dahmane S, Shen RY (2011). Modulation of the serotonin system by endocannabinoid signaling. Neuropharmacology.

[bib80] Han MH, Nestler EJ (2017). Neural substrates of depression and resilience. Neurotherapeutics.

[bib81] Hartung H, Brockmann MD, Pöschel B, De Feo V, Hanganu-Opatz IL (2016). Thalamic and entorhinal network activity differently modulates the functional development of Prefrontal-Hippocampal interactions. The Journal of Neuroscience.

[bib82] Hefner K, Holmes A (2007). Ontogeny of fear-, anxiety- and depression-related behavior across adolescence in C57BL/6J mice. Behavioural Brain Research.

[bib83] Heng L, Beverley JA, Steiner H, Tseng KY (2011). Differential developmental trajectories for CB1 cannabinoid receptor expression in limbic/associative and sensorimotor cortical Areas. Synapse.

[bib84] Hikida T, Jaaro-Peled H, Seshadri S, Oishi K, Hookway C, Kong S, Wu D, Xue R, Andradé M, Tankou S, Mori S, Gallagher M, Ishizuka K, Pletnikov M, Kida S, Sawa A (2007). Dominant-negative DISC1 transgenic mice display schizophrenia-associated phenotypes detected by measures translatable to humans. PNAS.

[bib85] Hill MN, McLaughlin RJ, Pan B, Fitzgerald ML, Roberts CJ, Lee TT, Karatsoreos IN, Mackie K, Viau V, Pickel VM, McEwen BS, Liu QS, Gorzalka BB, Hillard CJ (2011). Recruitment of prefrontal cortical endocannabinoid signaling by glucocorticoids contributes to termination of the stress response. Journal of Neuroscience.

[bib86] Hill RA, Kiss Von Soly S, Ratnayake U, Klug M, Binder MD, Hannan AJ, van den Buuse M (2014). Long-term effects of combined neonatal and adolescent stress on brain-derived neurotrophic factor and dopamine receptor expression in the rat forebrain. Biochimica Et Biophysica Acta (BBA) - Molecular Basis of Disease.

[bib87] Hiser J, Koenigs M (2018). The multifaceted role of the ventromedial prefrontal cortex in emotion, decision making, social cognition, and psychopathology. Biological Psychiatry.

[bib88] Holland FH, Ganguly P, Potter DN, Chartoff EH, Brenhouse HC (2014). Early life stress disrupts social behavior and prefrontal cortex parvalbumin interneurons at an earlier time-point in females than in males. Neuroscience Letters.

[bib89] Holt DJ, Coombs G, Zeidan MA, Goff DC, Milad MR (2012). Failure of neural responses to safety cues in schizophrenia. Archives of General Psychiatry.

[bib90] Howes OD, McCutcheon R, Owen MJ, Murray RM (2017). The role of genes, stress, and dopamine in the development of schizophrenia. Biological Psychiatry.

[bib91] Hu H, Gan J, Jonas P (2014). Fast-spiking, parvalbumin+ GABAergic interneurons: from cellular design to microcircuit function. Science.

[bib92] Hyman JM (2010). Working memory performance correlates with prefrontal-hippocampal theta interactions but not with prefrontal neuron firing rates. Frontiers in Integrative Neuroscience.

[bib93] Ibi D, Nagai T, Koike H, Kitahara Y, Mizoguchi H, Niwa M, Jaaro-Peled H, Nitta A, Yoneda Y, Nabeshima T, Sawa A, Yamada K (2010). Combined effect of neonatal immune activation and mutant DISC1 on phenotypic changes in adulthood. Behavioural Brain Research.

[bib94] Isaacson JS, Scanziani M (2011). How inhibition shapes cortical activity. Neuron.

[bib95] Izquierdo A, Brigman JL, Radke AK, Rudebeck PH, Holmes A (2017). The neural basis of reversal learning: an updated perspective. Neuroscience.

[bib96] Jia M, Travaglia A, Pollonini G, Fedele G, Alberini CM (2018). Developmental changes in plasticity, synaptic, Glia, and connectivity protein levels in rat medial prefrontal cortex. Learning & Memory.

[bib97] Johnson CM, Loucks FA, Peckler H, Thomas AW, Janak PH, Wilbrecht L (2016). Long-range orbitofrontal and amygdala axons show divergent patterns of maturation in the frontal cortex across adolescence. Developmental Cognitive Neuroscience.

[bib98] Johnson C, Wilbrecht L (2011). Juvenile mice show greater flexibility in multiple choice reversal learning than adults. Developmental Cognitive Neuroscience.

[bib99] Kalsbeek A, Voorn P, Buijs RM, Pool CW, Uylings HB (1988). Development of the dopaminergic innervation in the prefrontal cortex of the rat. The Journal of Comparative Neurology.

[bib100] Kawaguchi Y (1997). GABAergic cell subtypes and their synaptic connections in rat frontal cortex. Cerebral Cortex.

[bib101] Koike H, Arguello PA, Kvajo M, Karayiorgou M, Gogos JA (2006). Disc1 is mutated in the 129s6/SvEv strain and modulates working memory in mice. PNAS.

[bib102] Kolb B, Nonneman AJ, Singh RK (1974). Double dissociation of spatial impairments and perseveration following selective prefrontal lesions in rats. Journal of Comparative and Physiological Psychology.

[bib103] Kolb B, Mychasiuk R, Muhammad A, Li Y, Frost DO, Gibb R (2012). Experience and the developing prefrontal cortex. PNAS.

[bib104] Kolb B, Nonneman AJ (1978). Sparing of function in rats with early prefrontal cortex lesions. Brain Research.

[bib105] Kosaki Y, Watanabe S (2012). Dissociable roles of the medial prefrontal cortex, the anterior cingulate cortex, and the Hippocampus in behavioural flexibility revealed by serial reversal of three-choice discrimination in rats. Behavioural Brain Research.

[bib106] Kroon T, van Hugte E, van Linge L, Mansvelder HD, Meredith RM (2019). Early postnatal development of pyramidal neurons across layers of the mouse medial prefrontal cortex. Scientific Reports.

[bib107] Kvajo M, McKellar H, Arguello PA, Drew LJ, Moore H, MacDermott AB, Karayiorgou M, Gogos JA (2008). A mutation in mouse Disc1 that models a schizophrenia risk allele leads to specific alterations in neuronal architecture and cognition. PNAS.

[bib108] Latagliata EC, Patrono E, Puglisi-Allegra S, Ventura R (2010). Food seeking in spite of harmful consequences is under prefrontal cortical noradrenergic control. BMC Neuroscience.

[bib109] Lauzon NM, Bishop SF, Laviolette SR (2009). Dopamine D1 versus D4 receptors differentially modulate the encoding of salient versus nonsalient emotional information in the medial prefrontal cortex. Journal of Neuroscience.

[bib110] Lazaro MT, Taxidis J, Shuman T, Bachmutsky I, Ikrar T, Santos R, Marcello GM, Mylavarapu A, Chandra S, Foreman A, Goli R, Tran D, Sharma N, Azhdam M, Dong H, Choe KY, Peñagarikano O, Masmanidis SC, Rácz B, Xu X, Geschwind DH, Golshani P (2019). Reduced prefrontal synaptic connectivity and disturbed oscillatory population dynamics in the CNTNAP2 model of autism. Cell Reports.

[bib111] LeDoux JE (2009). Emotion circuits in the brain. Focus.

[bib112] Lee FH, Zai CC, Cordes SP, Roder JC, Wong AH (2013). Abnormal interneuron development in disrupted-in-schizophrenia-1 L100P mutant mice. Molecular Brain.

[bib113] Lee AT, Vogt D, Rubenstein JL, Sohal VS (2014). A class of GABAergic neurons in the prefrontal cortex sends long-range projections to the nucleus accumbens and elicits acute avoidance behavior. Journal of Neuroscience.

[bib114] Lee TT, Hill MN, Lee FS (2016). Developmental regulation of fear learning and anxiety behavior by endocannabinoids. Genes, Brain, and Behavior.

[bib115] Leslie CA, Robertson MW, Cutler AJ, Bennett JP (1991). Postnatal development of D 1 dopamine receptors in the medial prefrontal cortex, striatum and nucleus accumbens of normal and neonatal 6-hydroxydopamine treated rats: a quantitative autoradiographic analysis. Developmental Brain Research.

[bib116] Lesting J, Daldrup T, Narayanan V, Himpe C, Seidenbecher T, Pape HC (2013). Directional theta coherence in prefrontal cortical to amygdalo-hippocampal pathways signals fear extinction. PLOS ONE.

[bib117] Levitt P, Moore RY (1979). Development of the noradrenergic innervation of neocortex. Brain Research.

[bib118] Lewis DA (1997). Development of the prefrontal cortex during adolescence: insights into vulnerable neural circuits in schizophrenia. Neuropsychopharmacology.

[bib119] Lewis EM, Barnett JF, Freshwater L, Hoberman AM, Christian MS (2002). Sexual maturation data for crl sprague-dawley rats: criteria and confounding factors. Drug and Chemical Toxicology.

[bib120] Li W, Zhou Y, Jentsch JD, Brown RA, Tian X, Ehninger D, Hennah W, Peltonen L, Lönnqvist J, Huttunen MO, Kaprio J, Trachtenberg JT, Silva AJ, Cannon TD (2007). Specific developmental disruption of disrupted-in-schizophrenia-1 function results in schizophrenia-related phenotypes in mice. PNAS.

[bib121] Li S, Kim JH, Richardson R (2012). Updating memories: changing the involvement of the prelimbic cortex in the expression of an infant fear memory. Neuroscience.

[bib122] Li L, Shao J (1998). Restricted lesions to ventral prefrontal subareas block reversal learning but not visual discrimination learning in rats. Physiology & Behavior.

[bib123] Likhtik E, Stujenske JM, Topiwala MA, Harris AZ, Gordon JA (2014). Prefrontal entrainment of amygdala activity signals safety in learned fear and innate anxiety. Nature Neuroscience.

[bib124] Lin HC, Mao SC, Su CL, Gean PW (2009). The role of prefrontal cortex CB1 receptors in the modulation of fear memory. Cerebral Cortex.

[bib125] Liska A, Bertero A, Gomolka R, Sabbioni M, Galbusera A, Barsotti N, Panzeri S, Scattoni ML, Pasqualetti M, Gozzi A (2018). Homozygous loss of Autism-Risk gene CNTNAP2 results in reduced local and Long-Range prefrontal functional connectivity. Cerebral Cortex.

[bib126] Llorente-Berzal A, Terzian AL, di Marzo V, Micale V, Viveros MP, Wotjak CT (2015). 2-AG promotes the expression of conditioned fear via cannabinoid receptor type 1 on GABAergic neurons. Psychopharmacology.

[bib127] Lovelace JW, Vieira PA, Corches A, Mackie K, Korzus E (2014). Impaired fear memory specificity associated with deficient endocannabinoid-dependent long-term plasticity. Neuropsychopharmacology.

[bib128] Luo L, Callaway EM, Svoboda K (2018). Genetic dissection of neural circuits: a decade of progress. Neuron.

[bib129] Maddaloni G, Bertero A, Pratelli M, Barsotti N, Boonstra A, Giorgi A, Migliarini S, Pasqualetti M (2017). Development of serotonergic fibers in the Post-Natal mouse brain. Frontiers in Cellular Neuroscience.

[bib130] Manitt C, Eng C, Pokinko M, Ryan RT, Torres-Berrío A, Lopez JP, Yogendran SV, Daubaras MJ, Grant A, Schmidt ER, Tronche F, Krimpenfort P, Cooper HM, Pasterkamp RJ, Kolb B, Turecki G, Wong TP, Nestler EJ, Giros B, Flores C (2013). Dcc orchestrates the development of the prefrontal cortex during adolescence and is altered in psychiatric patients. Translational Psychiatry.

[bib131] Maren S, De Oca B, Fanselow MS (1994). Sex differences in hippocampal long-term potentiation (LTP) and pavlovian fear conditioning in rats: positive correlation between LTP and contextual learning. Brain Research.

[bib132] McCutcheon JE, White FJ, Marinelli M (2009). Individual differences in dopamine cell neuroadaptations following cocaine self-administration. Biological Psychiatry.

[bib133] McEwen BS, Morrison JH (2013). The brain on stress: vulnerability and plasticity of the prefrontal cortex over the life course. Neuron.

[bib134] McLaughlin RJ, Hill MN, Bambico FR, Stuhr KL, Gobbi G, Hillard CJ, Gorzalka BB (2012). Prefrontal cortical anandamide signaling coordinates coping responses to stress through a serotonergic pathway. European Neuropsychopharmacology.

[bib135] Millar JK, Wilson-Annan JC, Anderson S, Christie S, Taylor MS, Semple CA, Devon RS, St Clair DM, Muir WJ, Blackwood DH, Porteous DJ (2000). Disruption of two novel genes by a translocation co-segregating with schizophrenia. Human Molecular Genetics.

[bib136] Minatohara K, Akiyoshi M, Okuno H (2015). Role of Immediate-Early genes in synaptic plasticity and neuronal ensembles underlying the memory trace. Frontiers in Molecular Neuroscience.

[bib137] Mingote S, de Bruin JP, Feenstra MG (2004). Noradrenaline and dopamine efflux in the prefrontal cortex in relation to appetitive classical conditioning. Journal of Neuroscience.

[bib138] Miyamae T, Chen K, Lewis DA, Gonzalez-Burgos G (2017). Distinct physiological maturation of Parvalbumin-Positive neuron subtypes in mouse prefrontal cortex. The Journal of Neuroscience.

[bib139] Moin Afshar N, Keip AJ, Taylor JR, Lee D, Groman SM (2020). Reinforcement learning during adolescence in rats. The Journal of Neuroscience.

[bib140] Monteiro P, Feng G (2017). SHANK proteins: roles at the synapse and in autism spectrum disorder. Nature Reviews Neuroscience.

[bib141] Morilak DA (2012). Modulating the modulators: interaction of brain norepinephrine and cannabinoids in stress. Experimental Neurology.

[bib142] Mulder J, Aguado T, Keimpema E, Barabás K, Ballester Rosado CJ, Nguyen L, Monory K, Marsicano G, Di Marzo V, Hurd YL, Guillemot F, Mackie K, Lutz B, Guzmán M, Lu HC, Galve-Roperh I, Harkany T (2008). Endocannabinoid signaling controls pyramidal cell specification and long-range axon patterning. PNAS.

[bib143] Naisbitt S, Kim E, Tu JC, Xiao B, Sala C, Valtschanoff J, Weinberg RJ, Worley PF, Sheng M (1999). Shank, a novel family of postsynaptic density proteins that binds to the NMDA receptor/PSD-95/GKAP complex and cortactin. Neuron.

[bib144] Naneix F, Marchand AR, Di Scala G, Pape JR, Coutureau E (2012). Parallel maturation of goal-directed behavior and dopaminergic systems during adolescence. Journal of Neuroscience.

[bib145] Nishida M, Pearsall J, Buckner RL, Walker MP (2009). REM sleep, prefrontal Theta, and the consolidation of human emotional memory. Cerebral Cortex.

[bib146] Niwa M, Kamiya A, Murai R, Kubo K, Gruber AJ, Tomita K, Lu L, Tomisato S, Jaaro-Peled H, Seshadri S, Hiyama H, Huang B, Kohda K, Noda Y, O'Donnell P, Nakajima K, Sawa A, Nabeshima T (2010). Knockdown of DISC1 by in utero gene transfer disturbs postnatal dopaminergic maturation in the frontal cortex and leads to adult behavioral deficits. Neuron.

[bib147] Nonneman AJ, Voigt J, Kolb BE (1974). Comparisons of behavioral effects of hippocampal and prefrontal cortex lesions in the rat. Journal of Comparative and Physiological Psychology.

[bib148] Nussenbaum K, Hartley CA (2019). Reinforcement learning across development: what insights can we draw from a decade of research?. Developmental Cognitive Neuroscience.

[bib149] Ohta K, Miki T, Warita K, Suzuki S, Kusaka T, Yakura T, Liu JQ, Tamai M, Takeuchi Y (2014). Prolonged maternal separation disturbs the serotonergic system during early brain development. International Journal of Developmental Neuroscience.

[bib150] Otsuka I, Watanabe Y, Hishimoto A, Boku S, Mouri K, Shiroiwa K, Okazaki S, Nunokawa A, Shirakawa O, Someya T, Sora I (2015). Association analysis of the Cadherin13 gene with schizophrenia in the japanese population. Neuropsychiatric Disease and Treatment.

[bib151] Parolaro D, Realini N, Vigano D, Guidali C, Rubino T (2010). The endocannabinoid system and psychiatric disorders. Experimental Neurology.

[bib152] Pattwell SS, Bath KG, Casey BJ, Ninan I, Lee FS (2011). Selective early-acquired fear memories undergo temporary suppression during adolescence. PNAS.

[bib153] Peñagarikano O, Abrahams BS, Herman EI, Winden KD, Gdalyahu A, Dong H, Sonnenblick LI, Gruver R, Almajano J, Bragin A, Golshani P, Trachtenberg JT, Peles E, Geschwind DH (2011). Absence of CNTNAP2 leads to epilepsy, neuronal migration abnormalities, and core autism-related deficits. Cell.

[bib154] Phelan K, McDermid HE (2012). The 22q13.3 deletion syndrome (Phelan-McDermid syndrome). Molecular Syndromology.

[bib155] Phillips AG, Ahn S, Floresco SB (2004). Magnitude of dopamine release in medial prefrontal cortex predicts accuracy of memory on a delayed response task. Journal of Neuroscience.

[bib156] Piekarski DJ, Johnson CM, Boivin JR, Thomas AW, Lin WC, Delevich K, M Galarce E, Wilbrecht L (2017a). Does puberty mark a transition in sensitive periods for plasticity in the associative neocortex?. Brain Research.

[bib157] Piekarski DJ, Boivin JR, Wilbrecht L (2017b). Ovarian hormones organize the maturation of inhibitory neurotransmission in the frontal cortex at puberty onset in female mice. Current Biology.

[bib158] Pokinko M, Grant A, Shahabi F, Dumont Y, Manitt C, Flores C (2017). Dcc haploinsufficiency regulates dopamine receptor expression across postnatal lifespan. Neuroscience.

[bib159] Poliak S, Gollan L, Martinez R, Custer A, Einheber S, Salzer JL, Trimmer JS, Shrager P, Peles E (1999). Caspr2, a new member of the neurexin superfamily, is localized at the juxtaparanodes of myelinated axons and associates with K+ channels. Neuron.

[bib160] Radnikow G, Feldmeyer D (2018). Layer- and cell Type-Specific modulation of excitatory neuronal activity in the neocortex. Frontiers in Neuroanatomy.

[bib161] Ramsaran AI, Schlichting ML, Frankland PW (2019). The ontogeny of memory persistence and specificity. Developmental Cognitive Neuroscience.

[bib162] Rao N, Northoff G, Tagore A, Rusjan P, Kenk M, Wilson A, Houle S, Strafella A, Remington G, Mizrahi R (2019). Impaired prefrontal cortical dopamine release in schizophrenia during a cognitive task: a [11C]FLB 457 positron emission tomography study. Schizophrenia Bulletin.

[bib163] Rapanelli M, Frick LR, Pittenger C (2017). The role of interneurons in autism and tourette syndrome. Trends in Neurosciences.

[bib164] Rebello TJ, Yu Q, Goodfellow NM, Caffrey Cagliostro MK, Teissier A, Morelli E, Demireva EY, Chemiakine A, Rosoklija GB, Dwork AJ, Lambe EK, Gingrich JA, Ansorge MS (2014). Postnatal day 2 to 11 constitutes a 5-HT-sensitive period impacting adult mPFC function. Journal of Neuroscience.

[bib165] Renard J, Szkudlarek HJ, Kramar CP, Jobson CEL, Moura K, Rushlow WJ, Laviolette SR (2017). Adolescent THC exposure causes enduring prefrontal cortical disruption of GABAergic inhibition and dysregulation of Sub-Cortical dopamine function. Scientific Reports.

[bib166] Reyes BA, Szot P, Sikkema C, Cathel AM, Kirby LG, Van Bockstaele EJ (2012). Stress-induced sensitization of cortical adrenergic receptors following a history of cannabinoid exposure. Experimental Neurology.

[bib167] Reynolds LM, Makowski CS, Yogendran SV, Kiessling S, Cermakian N, Flores C (2015). Amphetamine in adolescence disrupts the development of medial prefrontal cortex dopamine connectivity in a DCC-dependent manner. Neuropsychopharmacology.

[bib168] Reynolds LM, Pokinko M, Torres-Berrío A, Cuesta S, Lambert LC, Del Cid Pellitero E, Wodzinski M, Manitt C, Krimpenfort P, Kolb B, Flores C (2018). DCC receptors drive prefrontal cortex maturation by determining dopamine axon Targeting in Adolescence. Biological Psychiatry.

[bib169] Rinetti-Vargas G, Phamluong K, Ron D, Bender KJ (2017). Periadolescent maturation of GABAergic hyperpolarization at the axon initial segment. Cell Reports.

[bib170] Rivero O, Sich S, Popp S, Schmitt A, Franke B, Lesch KP (2013). Impact of the ADHD-susceptibility gene CDH13 on development and function of brain networks. European Neuropsychopharmacology.

[bib171] Rivero O, Selten MM, Sich S, Popp S, Bacmeister L, Amendola E, Negwer M, Schubert D, Proft F, Kiser D, Schmitt AG, Gross C, Kolk SM, Strekalova T, van den Hove D, Resink TJ, Nadif Kasri N, Lesch KP (2015). Cadherin-13, a risk gene for ADHD and comorbid disorders, impacts GABAergic function in Hippocampus and cognition. Translational Psychiatry.

[bib172] Rodgers RJ, Boullier E, Chatzimichalaki P, Cooper GD, Shorten A (2002). Contrasting phenotypes of C57BL/6JOlaHsd, 129s2/SvHsd and 129/SvEv mice in two exploration-based tests of anxiety-related behaviour. Physiology & Behavior.

[bib173] Sala C, Piëch V, Wilson NR, Passafaro M, Liu G, Sheng M (2001). Regulation of dendritic spine morphology and synaptic function by shank and homer. Neuron.

[bib174] Schipper P, Brivio P, de Leest D, Madder L, Asrar B, Rebuglio F, Verheij MMM, Kozicz T, Riva MA, Calabrese F, Henckens MJAG, Homberg JR (2019). Impaired fear extinction recall in serotonin transporter knockout rats is transiently alleviated during adolescence. Brain Sciences.

[bib175] Schofield CM, Hsu R, Barker AJ, Gertz CC, Blelloch R, Ullian EM (2011). Monoallelic deletion of the microRNA biogenesis gene Dgcr8 produces deficits in the development of excitatory synaptic transmission in the prefrontal cortex. Neural Development.

[bib176] Schubert D, Martens GJ, Kolk SM (2015). Molecular underpinnings of prefrontal cortex development in rodents provide insights into the etiology of neurodevelopmental disorders. Molecular Psychiatry.

[bib177] Shapiro LP, Parsons RG, Koleske AJ, Gourley SL (2017). Differential expression of cytoskeletal regulatory factors in the adolescent prefrontal cortex: implications for cortical development. Journal of Neuroscience Research.

[bib178] Shen S, Lang B, Nakamoto C, Zhang F, Pu J, Kuan SL, Chatzi C, He S, Mackie I, Brandon NJ, Marquis KL, Day M, Hurko O, McCaig CD, Riedel G, St Clair D (2008). Schizophrenia-related neural and behavioral phenotypes in transgenic mice expressing truncated Disc1. Journal of Neuroscience.

[bib179] Simon NW, Moghaddam B (2015). Neural processing of reward in adolescent rodents. Developmental Cognitive Neuroscience.

[bib180] Sohal VS, Zhang F, Yizhar O, Deisseroth K (2009). Parvalbumin neurons and gamma rhythms enhance cortical circuit performance. Nature.

[bib181] Sohal VS, Rubenstein JLR (2019). Excitation-inhibition balance as a framework for investigating mechanisms in neuropsychiatric disorders. Molecular Psychiatry.

[bib182] Soiza-Reilly M, Meye FJ, Olusakin J, Telley L, Petit E, Chen X, Mameli M, Jabaudon D, Sze JY, Gaspar P (2019). SSRIs target prefrontal to raphe circuits during development modulating synaptic connectivity and emotional behavior. Molecular Psychiatry.

[bib183] Somogyi P, Freund TF, Cowey A (1982). The axo-axonic interneuron in the cerebral cortex of the rat, cat and monkey. Neuroscience.

[bib184] Spear LP (2000). The adolescent brain and age-related behavioral manifestations. Neuroscience & Biobehavioral Reviews.

[bib185] Stoya G, Redies C, Schmid-Hertel N (2014). Inversion of layer-specific cadherin expression profiles and maintenance of cytoarchitectonic Areas in the allocortex of the reeler mutant mouse. Journal of Comparative Neurology.

[bib186] Strauss KA, Puffenberger EG, Huentelman MJ, Gottlieb S, Dobrin SE, Parod JM, Stephan DA, Morton DH (2006). Recessive symptomatic focal epilepsy and mutant Contactin-Associated Protein-like 2. New England Journal of Medicine.

[bib187] Sun Q, Li X, Ren M, Zhao M, Zhong Q, Ren Y, Luo P, Ni H, Zhang X, Zhang C, Yuan J, Li A, Luo M, Gong H, Luo Q (2019). A whole-brain map of long-range inputs to GABAergic interneurons in the mouse medial prefrontal cortex. Nature Neuroscience.

[bib188] Tan H, Lauzon NM, Bishop SF, Bechard MA, Laviolette SR (2010). Integrated cannabinoid CB1 receptor transmission within the amygdala-prefrontal cortical pathway modulates neuronal plasticity and emotional memory encoding. Cerebral Cortex.

[bib189] Tarazi FI, Baldessarini RJ (2000). Comparative postnatal development of dopamine D(1), D(2) and D(4) receptors in rat forebrain. International Journal of Developmental Neuroscience.

[bib190] Travaglia A, Bisaz R, Sweet ES, Blitzer RD, Alberini CM (2016). Infantile amnesia reflects a developmental critical period for hippocampal learning. Nature Neuroscience.

[bib191] Uhlhaas PJ, Singer W (2010). Abnormal neural oscillations and synchrony in schizophrenia. Nature Reviews Neuroscience.

[bib192] Van Eden CG, Uylings HB (1985). Cytoarchitectonic development of the prefrontal cortex in the rat. The Journal of Comparative Neurology.

[bib193] Vander Weele CM, Siciliano CA, Matthews GA, Namburi P, Izadmehr EM, Espinel IC, Nieh EH, Schut EHS, Padilla-Coreano N, Burgos-Robles A, Chang CJ, Kimchi EY, Beyeler A, Wichmann R, Wildes CP, Tye KM (2018). Dopamine enhances signal-to-noise ratio in cortical-brainstem encoding of aversive stimuli. Nature.

[bib194] Ventura R, Cabib S, Alcaro A, Orsini C, Puglisi-Allegra S (2003). Norepinephrine in the prefrontal cortex is critical for amphetamine-induced reward and mesoaccumbens dopamine release. The Journal of Neuroscience.

[bib195] Ventura R, Alcaro A, Puglisi-Allegra S (2005). Prefrontal cortical norepinephrine release is critical for morphine-induced reward, reinstatement and dopamine release in the nucleus accumbens. Cerebral Cortex.

[bib196] Ventura R, Latagliata EC, Morrone C, La Mela I, Puglisi-Allegra S (2008). Prefrontal norepinephrine determines attribution of "high" motivational salience. PLOS ONE.

[bib197] Ventura R, Coccurello R, Andolina D, Latagliata EC, Zanettini C, Lampis V, Battaglia M, D'Amato FR, Moles A (2013). Postnatal aversive experience impairs sensitivity to natural rewards and increases susceptibility to negative events in adult life. Cerebral Cortex.

[bib198] Vizi ES, Zsilla G, Caron MG, Kiss JP (2004). Uptake and release of norepinephrine by serotonergic terminals in norepinephrine transporter knock-out mice: implications for the action of selective serotonin reuptake inhibitors. Journal of Neuroscience.

[bib199] Vogt D, Cho KKA, Shelton SM, Paul A, Huang ZJ, Sohal VS, Rubenstein JLR (2018). Mouse Cntnap2 and human CNTNAP2 ASD alleles cell autonomously regulate PV+ cortical interneurons. Cerebral Cortex.

[bib200] Volk DW, Lewis DA (2013). Prenatal ontogeny as a susceptibility period for cortical GABA neuron disturbances in schizophrenia. Neuroscience.

[bib201] Wang Y, Dye CA, Sohal V, Long JE, Estrada RC, Roztocil T, Lufkin T, Deisseroth K, Baraban SC, Rubenstein JL (2010). Dlx5 and Dlx6 regulate the development of parvalbumin-expressing cortical interneurons. Journal of Neuroscience.

[bib202] Watt MJ, Roberts CL, Scholl JL, Meyer DL, Miiller LC, Barr JL, Novick AM, Renner KJ, Forster GL (2014). Decreased prefrontal cortex dopamine activity following adolescent social defeat in male rats: role of dopamine D2 receptors. Psychopharmacology.

[bib203] Wellman CL, Izquierdo A, Garrett JE, Martin KP, Carroll J, Millstein R, Lesch KP, Murphy DL, Holmes A (2007). Impaired stress-coping and fear extinction and abnormal corticolimbic morphology in serotonin transporter knock-out mice. Journal of Neuroscience.

[bib204] Willing J, Cortes LR, Brodsky JM, Kim T, Juraska JM (2017). Innervation of the medial prefrontal cortex by tyrosine hydroxylase immunoreactive fibers during adolescence in male and female rats. Developmental Psychobiology.

[bib205] Wiltgen BJ, Sanders MJ, Behne NS, Fanselow MS (2001). Sex differences, context preexposure, and the immediate shock deficit in pavlovian context conditioning with mice. Behavioral Neuroscience.

[bib206] Yizhar O, Fenno LE, Prigge M, Schneider F, Davidson TJ, O'Shea DJ, Sohal VS, Goshen I, Finkelstein J, Paz JT, Stehfest K, Fudim R, Ramakrishnan C, Huguenard JR, Hegemann P, Deisseroth K (2011). Neocortical excitation/inhibition balance in information processing and social dysfunction. Nature.

[bib207] Yu Q, Liu YZ, Zhu YB, Wang YY, Li Q, Yin DM (2019). Genetic labeling reveals temporal and spatial expression pattern of D2 dopamine receptor in rat forebrain. Brain Structure and Function.

